# ScanNet: Single-cell annotation informed by transcriptional regulation Network via iterative heterogeneous graph learning

**DOI:** 10.1371/journal.pcbi.1014602

**Published:** 2026-08-06

**Authors:** Yongyu Long, Wenhao Zhang, Lan Cao, Xiaobing Huang, Ying Wang

**Affiliations:** 1 Department of Automation, National Institute for Data Science in Health and Medicine, State Key Laboratory of Mariculture Breeding, Xiamen Key Laboratory of Big Data Intelligent Analysis and Decision, Xiamen University, Xiamen, Fujian, China; 2 Department of Medical Oncology, Fuzhou First Hospital Affiliated with Fujian Medical University, Fuzhou, Fujian, China; Western University, CANADA

## Abstract

Accurate annotation of cell types in single-cell transcriptome sequencing (scRNA-seq) data is critical for understanding cellular identities. The transcriptional regulatory networks (TRNs), which map the regulatory relationships between transcription factors (TFs) and their target genes (TGs), capture the molecular dependencies underlying transcriptional programs. However, most existing cell type annotation methods do not fully exploit this regulatory information. Therefore, we introduce **ScanNet**, a Single cell annotation method informed by transcriptional regulation Network, to integrate prior knowledge of TRN into data of gene expression and capture the cell-type-specific characteristics underlying TRN mechanism. TRN can be naturally represented as heterogeneous graphs consisting of two regulatory elements, TFs and TGs connected by directed edges, thereby encoding the regulatory dependencies that shape transcriptional programs and ultimately determine cellular identity. To leverage this structure, ScanNet introduces an iterative heterogeneous graph convolutional framework that learns both local and global cellular embeddings through a dual-channel encoder. The Regulation-level Encoder applies iterative heterogeneous graph convolution to capture local TF-TG regulatory interactions within TRN, while the Expression-level Encoder learns global cellular transcriptional states. By integrating the multiple-view representations, ScanNet can accurately annotate cell types. Comprehensive evaluations across eight scRNA-seq datasets spanning different species, sample scales, and sequencing platforms demonstrate that ScanNet consistently outperforms ten state-of-the-art cell type annotation methods. By embedding prior TRN structures into a heterogeneous graph, ScanNet also achieves robust performance in cross-platform cell type annotation and in identifying novel cell types under constrained structural information. Moreover, the ScanNet framework can be flexibly transferred to single-cell ATAC-seq (scATAC-seq) data by mapping chromatin accessibility to gene level, where it achieves superior performance compared to existing annotation tools. Overall, ScanNet is a scalable, transferable, and mechanistically informed framework for accurate cell type annotation across diverse single-cell data modalities.

## Author summary

Accurate cell type annotation is essential for deciphering cellular heterogeneity. Most existing annotating methods are based on gene expression profile from scRNA-seq data. However, behind the observed gene expression profile, gene regulation is the essential mechanism to drive the transcriptional progress, which offer more intrinsic biological insight. While most current annotation approaches don’t incorporate regulatory information, limiting their capacity to capture the mechanistic basis of cell states. Therefore, we incorporate transcriptional regulatory networks (TRNs) as prior knowledge to assist more accurate cell type annotation. We introduce ScanNet, an iterative heterogenous graph learning framework that incorporates TRNs as both prior knowledge and structural constraints to capture biologically interpretable, cell-type-specific characteristics. In benchmark evaluations with ten baselines on multiple datasets, ScanNet shows (1) superior annotation performance on scRNA-seq data, (2) robust performance across seven sequencing platforms, (3) capability to identify novel cellular populations beyond existing annotations, and (4) effective transferability to scATAC‑seq data.

## Introduction

Single-cell RNA sequencing (scRNA-seq) techniques enable the measurement of gene expression profiles at single-cell resolution, providing a powerful tool for revealing cellular heterogeneity and transcriptional states [1–4]. This cellular resolution necessitates accurate cell type annotation to properly interpret cell-type-specific molecular mechanisms [5–7]. However, the inherently sparse, high dropout rate and noisy nature of scRNA-seq data poses significant challenges to cell annotation.

Traditional manual approaches map marker genes with unsupervised clusters and annotate cells manually. While offering high biological interpretability, manual cell annotation is time-consuming, subjective, and poorly scalable, making it unreliable for large and complex single-cell datasets. To improve efficiency, computational methods start using similarity metrics to assess resemblance between query cells and reference datasets, automatically annotating the queried cells. For example, scmap [8] maps the cells with reference dataset based on options of Cosine similarity, Pearson correlation and Spearman correlation. CHETAH [9] constructs a hierarchical classification tree, where Spearman correlation and silhouette scores are used to assess the similarity between cells. However, these similarity-based approaches are susceptible to batch effects and the quality of reference datasets, showing fluctuated performance.

With the development of machine learning and deep learning, more supervised methods have emerged to improve annotation accuracy. These methods typically rely on feature selection or feature mapping, which subsequently serve as the basis for classification. For example, scID [10] selects feature genes by employing Fisher’s linear discriminant analysis to estimate gene weights; CaSTLe [11] and XGBoost [12] rely on tree-based ensemble methods; and scPred [13] employs a support vector machine to project gene expressions into a latent feature space. Furthermore, superCT [14] and ACTINN [15] utilize multi-layer neural networks to embed gene expressions, thereby reducing the need for feature selection; scGCN [16] constructs cell similarity graph and uses graph convolutional network (GCN) to facilitate cross-dataset cell annotation; scGraphformer [17] combines GCN with Transformer to uncover cellular heterogeneity.

However, due to individual variability and sequencing-related technical noise, the methods that rely solely on expression data still lack generalizability across datasets. Accordingly, biological networks are introduced as structural priors to uncover the biological essence and enhance model generalization. sigGCN [18] incorporates protein interaction network from String [19] by utilizing Chebyshev Polynomial-based variant of GCN to learn cell heterogeneity representations. Similarly, scGraph [20] integrates protein interaction networks by GraphSAGE [21]. scRGCL [22] utilizes a residual GCN and contrastive learning to optimize cell feature representation and enhance the discrimination of different cell types. While these methods attempt to capture molecular dependencies among genes, correlation-based interactions still fail to encapsulate causal regulatory relationships. The transcriptional regulatory network (TRN), in contrast, represents the directional relationships from transcription factors (TFs) to target genes (TGs) that drive gene expression, thereby describing the essential mechanisms of cellular heterogeneity.

Therefore, in this study, we propose **ScanNet**, a Single cell annotation method informed by transcriptional regulation Network via iterative heterogeneous graph learning. TRN can be naturally represented by a heterogeneous graph with two types of regulatory elements, where TFs modulate TG expressions by binding to promoter or enhancer regions, either activating or repressing transcription [23–27]. To leverage this structure, ScanNet introduces an iterative heterogeneous graph convolutional (iHGCN) framework that learns both local and global cellular embeddings through a dual-channel encoder consisting of a Regulation-level Encoder and an Expression-level Encoder. Specifically, the Regulation-level Encoder applies iHGCN to capture local TF-TG molecular interactions within cells and learn biologically-informed Regulation-level Embeddings that fusing regulatory information and gene expression. While the Expression-level Encoder learns global cellular transcriptional states from overall cellular expression by neural networks. By integrating these two complementary representations, ScanNet can accurately annotate cell types.

We conduct extensive experiments to demonstrate the superiority of our method over state-of-the-art baselines under four scenarios. Firstly, evaluations across eight scRNA-seq datasets that varied from species, sample scales, and sequencing platforms demonstrate the superiority of ScanNet over baseline methods in basic annotation task. By introducing TRN as basis structure and prior constraint, ScanNet achieves robust annotation performance in cross-platform datasets despite technical noise and batch effects. In addition, ScanNet can automatically identify previously unseen cell types, also referred as novel types through entropy-based uncertainty quantification in open-world scenario. Finally, the ScanNet framework can be flexibly transferred to single-cell ATAC-seq data by mapping chromatin accessibility to gene level, where it achieves superior performance compared to existing annotation tools. Overall, by integrating cellular transcriptional states with underlying regulatory mechanisms through iHGCN, ScanNet not only effectively captures the fundamental signatures of cell states but also deeply utilizes the causal linkages between transcriptional regulation and gene expression dynamics, providing regulatory perspective-based insights that advance our understanding of the mechanistic drivers of cellular heterogeneity.

## Results

### The framework of ScanNet

**Fig 1:**
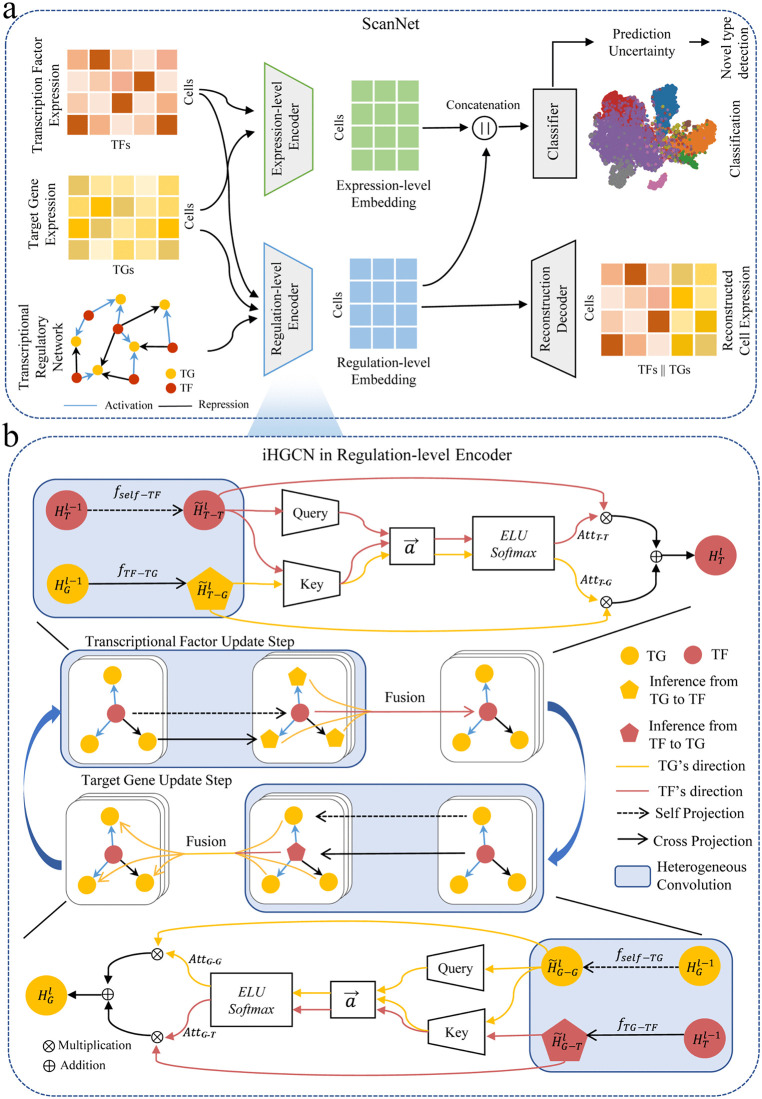
Framework of ScanNet. (a) Overall architecture of ScanNet. ScanNet takes the expressions of TF and TG, and a reference TRN as input. ScanNet consists of a dual-channel encoder, a Regulation-level Encoder and an Expression-level Encoder, a Reconstruction Decoder and a Classifier. **(b)** Details of the iHGCN in the Regulation-level Encoder. The iHGCN captures regulatory dependencies by alternately updating TF and TG representations through heterogeneous message passing on the TRN, involving TF update and TG update steps.

ScanNet is a new solution driven by both regulatory mechanisms and data characteristics for scRNA-seq data annotation (Fig 1a). ScanNet takes scRNA-seq data as input and reference TRN as basis regulatory structure to construct a heterogeneous graph that simultaneously models gene expression profiles and regulatory mechanisms. Specifically, the reference TRN is modeled as a heterogeneous graph by distinguishing TF and TG nodes and their regulatory interactions. The regulatory intensity is quantified as edge weights, with 1 assigned to activating regulation and −1 to repressive regulation. The TRN further guides the selection of relevant genes from the scRNA-seq data, yielding TF and TG expression features for model input (S1 Text). With the prior constraint, ScanNet is able to capture cellular heterogeneity from both molecular mechanisms and transcriptional state through a dual-channel encoder with iterative graph convolution. The dual-channel encoder comprises a Regulation-level Encoder, which is responsible to extract causal regulatory dependencies at the molecular level through iHGCN (Fig 1b), and an Expression-level Encoder that learns global transcriptional states at the cellular level. By embedding regulatory dependencies, ScanNet generates biologically-informed cellular representations that not only enhance annotation accuracy but also improve generalization and applicability to diverse annotation tasks, like cross-platform annotation, novel cell type discovery, and transferability to single-cell ATAC-seq (scATAC-seq).

### Dataset description and experimental design

We evaluate the performance of ScanNet against ten baseline methods across eight scRNA-seq datasets for the cell annotation task. Benchmark datasets include four human datasets, the paired multimodal PBMC dataset [28], Muraro dataset [29] derived from human pancreas, BMMC dataset [30] from bone marrow and Immune dataset from multi-tissue immune system [31], as well as four mouse datasets, BaronMouse dataset [32] from mouse pancreas, ChenMouse dataset [33] and AMB dataset [34] from mouse visual cortex, and TM dataset [35] from mouse musculus. For each benchmark dataset, cell-type labels are manually curated by domain experts and treated as the gold standard for a unified evaluation of automated annotation methods. Although datasets are derived from different species, the reference TRN [24] is unified which collects TF-TG regulations from multiple cell types and tissues, covering both human and mouse data. These datasets vary from tissues, sequencing platforms, species as well as the number of TFs, TGs, cells and cell types, as shown in Table 1. This diversity ensures a comprehensive assessment of performance across various biological contexts and technical challenges in cell classification. To mitigate the impact of extremely rare cell types on classification performance, scRNA-seq-based cell annotation experiments filter out cell types with fewer than 10 cells. The excluded rare cell types can subsequently serve as novel cell types to evaluate the model’s novel type detection capability.

**Table 1:** Description of the eight scRNA-seq benchmark datasets.

Dataset	Sequencing Platform	Specie	Tissue	TF	TG	Cell	Cell Type
PBMC	10X Chromium v3	Human	Peripheral blood mononuclear cell	266	684	9,631	19(19)
Muraro	CEL-seq2	Human	Pancreas	106	798	2,122	9(8)
BMMC	10X Chromium	Human	Bone marrow mononuclear cell	213	673	69,249	22(22)
Immune	10X-based integration	Human	Multi-tissue immune system	460	917	329,762	35(35)
AMB	SMART-Seq v4	Mouse	Primary mouse visual cortex	52	362	12,832	22(16)
TM	SMART-Seq2	Mouse	Whole mouse musculus	90	979	54,865	55(55)
BaronMouse	inDrop	Mouse	Pancreas	112	1,006	1,886	13(10)
ChenMouse	SNARE-seq	Mouse	Mouse cerebral cortex	245	657	9,190	22(22)

Note: the numbers outside the brackets indicate the total number of cell types; the numbers in brackets denote those with more than 10 cells*.*

scRNA-seq data generated by different sequencing platforms exhibit systematic technical variations that pose challenges for cell annotation [36]. To evaluate the generalization of ScanNet across different sequencing platforms, another dataset (PBMCbench [37]) containing scRNA-seq data from seven sequencing platforms is introduced (S1 Table). Specifically, we design cross-validation experiments in which ScanNet is trained on one platform and tested on the rest six platforms, finally getting seven groups of experimental results. In addition, the batch-correction capability of ScanNet is evaluated by comparison with six baseline methods that support batch correction.

In a real-world scenario, the most models would assign the cells from previously unknown cell types to known types wrongly [38,39]. To evaluate ScanNet’s annotation capability in open-world scenario, we apply the pre-trained ScanNet model (trained on known cell types only) to new test datasets containing unknown cell types, also referred as novel types. Specifically, rare cell types with fewer than 10 cells, which are removed from the BaronMouse and AMB datasets during the initial training phase (Table 1), are reintroduced into the test datasets as new test datasets to simulate scenarios encountering novel types.

scATAC-seq provides insights into cellular heterogeneity by measuring chromatin accessibility, where the opening and closing of chromatin regions determine regulatory protein access and thereby influence gene expression initiation [40]. Given that scATAC-seq offers insights into deeper regulatory mechanisms through chromatin state profiling, we attempt to transfer the ScanNet framework to cell annotation on scATAC-seq data. Existing scATAC-seq classification tools [41] typically convert peak matrixes into gene score matrixes for downstream analysis, therefore we employ Signac [42] to transform chromatin accessibility signals into gene score matrixes that substitute for gene expression matrices in the ScanNet framework (S2 Text and S1 Fig). To evaluate the transferability of the ScanNet framework to scATAC-seq data, we use the scATAC-seq data from PBMC dataset [28]. Since this scATAC-seq data is paired with scRNA-seq data from the same PBMC dataset, we can compare the annotation performance of ScanNet across different modalities.

Given that benchmark datasets exhibit substantial class imbalance, we employ stratified sampling [43] to split each cell type into training and test sets at a 7:3 ratio, in which samples are drawn proportionally from each types to ensure that the overall sample accurately reflects the composition of the population. In addition, four evaluation metrics including Area Under the Precision-Recall Curve (AUPRC), F1, Precision and Recall, are employed to benchmark performance (S3 Text).

To extensively evaluate the performance of ScanNet in cell annotation, we benchmark it against ten baseline methods. These methods can be categorized into three groups: (i) reference mapping method (Seurat), (ii) deep learning-based methods (ACTINN [15], scAGN [44], scGCN [45], scGraphformer [17], scANVI [46]), and (iii) biological knowledge-informed methods (e.g., sigGCN [18], GEDFN [47], scGraph [20], and scRGCL [22]). Deep learning models are solely based on transcriptional expression profiles, while biological knowledge informed methods incorporate additional gene interaction networks as prior knowledge, ensuring that biological knowledge is explicitly embedded into cellular features. The protein-protein interaction (PPI) networks used by sigGCN, scGraph, and scRGCL are derived from the STRING database [19], whereas GEDFN uses a network obtained from the HINT database [48]. In contrast, ScanNet leverages transcriptional regulatory relationships between TFs and TGs encoded in a reference TRN as prior structural information. Among all compared methods, only scGCN, scAGN, Seurat, and scANVI adopt a semi-supervised learning paradigm, in which cell-type labels are transferred from a reference dataset to a query dataset for cell annotation. The remaining methods are fully supervised. The other benchmark methods are supervised learning models. All baseline models are implemented with their default hyperparameters.

### ScanNet outperforms baselines in cell type annotation

**Fig 2:**
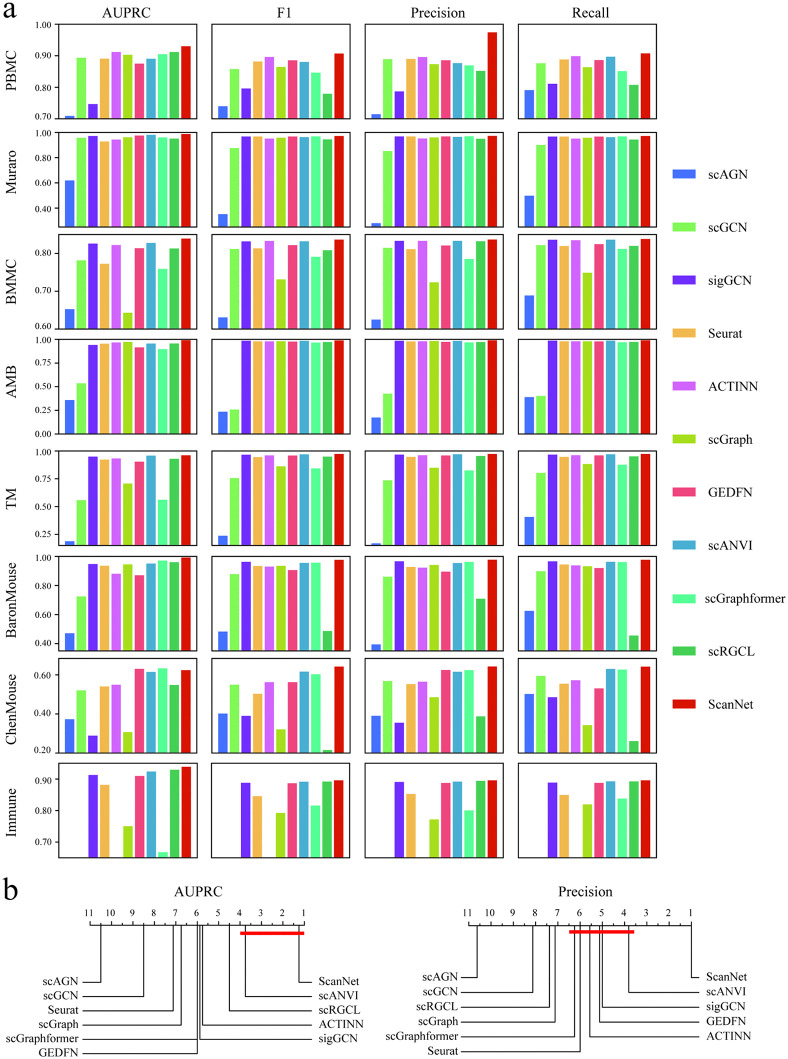
Performance evaluation of ScanNet on cell type annotation. **(a)** Comparison with ten methods across eight benchmark datasets on four evaluation metrics: AUPRC, F1 score, Precision, and Recall. **(b)** Critical difference diagrams for the two metrics: AUPRC and Precision. Methods connected by red are not significantly different in performance according to the Nemenyi test.

We benchmark ScanNet against ten baseline methods on the eight benchmark datasets spanning different species, tissues and sizes. ScanNet performs better than all baselines in terms of F1 score and Precision (Fig 2a and S2 Table). ScanNet achieves an F1 score of 0.9762 and Precision of 0.9776 in BaronMouse dataset, outperforming the second-best baseline by 1.40% and 2.06% respectively. In PBMC dataset, the precision of ScanNet is 0.9742, while those for the best three baselines (ACTINN, Seurat and scGCN) are 0.8959, 0.8899 and 0.8891. For AUPRC and Recall, ScanNet outperforms all baselines on all datasets. In AMB dataset, ScanNet is superior to the top two baseline methods, scGraph and ACTINN, with 1.83% and 2.41% improvement in terms of AUPRC. Both the ChenMouse and BMMC datasets are considered challenging due to the high similarity among cell types. Although ScanNet achieves a slightly lower AUPRC than scGraphformer in the ChenMouse dataset, it outperforms scGraphformer in terms of F1 score and Precision. Moreover, ScanNet achieves the best performance across all evaluation metrics on the BMMC dataset. These results indicate that ScanNet is also well suited for complex datasets.

In order to quantify the comparison with statistical rigor, we employed the Nemenyi test [49] and visualized the results using Critical Difference (CD) diagrams [50] at confidence level = 0.95, following the procedure established by Zhang et al. [40]. The CD diagrams are designed to determine whether one algorithm achieves significantly better performance than others through statistical testing. In the CD diagram, the horizontal axis denotes the mean rank of each method across all datasets, and red lines connect methods whose differences are not statistically significant. ScanNet ranks first across all classification metrics (Figs 2b and S2). Although its AUPRC performance is comparable to that of scANVI, ScanNet achieves a markedly better average rank (1.28 vs. 3.85). Moreover, ScanNet significantly outperforms all competing methods, including scANVI, in term of Precision. Finally, although ScanNet does not achieve the highest AUPRC in the ChenMouse dataset, it exhibits statistically significant superiority over scGraphformer in overall AUPRC across datasets.

**Fig 3:**
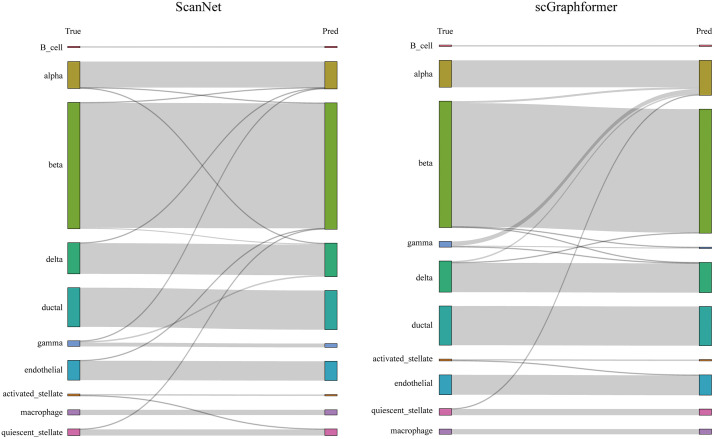
Comparison of cell type annotation performance between ScanNet and scGraphformer on the BaronMouse dataset. Sankey diagrams illustrate the correspondence between ground-truth (left) and predicted (right) cell type annotations on the test set. Each node represents a cell type, and the width of each connecting link reflects the number of cells assigned to the corresponding true-predicted pair. ScanNet demonstrates higher annotation accuracy with fewer cross-type misclassifications compared to scGraphformer.

scRNA-seq datasets usually exhibit obvious imbalance in types (S3 Fig). The cell numbers in different cell types are illustrated by the bar heights spanning from 894 beta cells to 10 B cells in BaronMouse dataset (Fig 3). This uneven distribution of sample numbers across cell types creates a typical scenario where model tends to learn the feature distribution of types with large sample size (e.g., beta cells) while neglecting the learning of minority class types (e.g., gamma cells). The Sankey diagrams (Fig 3) visualizes the correspondence between predicted and true cell types. We illustrate the results on BaronMouse dataset, which has the most pronounced cell type imbalance, and compare ScanNet with scGraphformer, the strongest baseline method. Nodes on the left represent ground-truth annotations, while nodes on the right denote model predictions. The width of each connecting link is proportional to the number of cells in each true-predicted type pair, which highlights that, for the minority gamma cell population, ScanNet demonstrates more direct correspondences with thicker Sankey flows between true and predicted labels, yielding a recall of 66.67%, whereas scGraphformer attains only 16.67%. Moreover, ScanNet attains a precision of 94.38% on alpha cells, surpassing scGraphformer’s 77.03%. This result reveals that ScanNet achieves superior accuracy on minority cell types, demonstrating its enhanced ability to mitigate recognition bias toward majority classes while maintaining robust overall predictive performance.

In addition to benchmark datasets of varying complexity and class imbalance, we further evaluate ScanNet on a large-scale human immune atlas dataset containing 329,762 cells to assess its performance on large-scale atlases. Because large-scale single-cell datasets introduce significant computational challenges, particularly for methods that rely on constructing global cell-cell graphs, as the number of cells increases, the cost of nearest-neighbor search, graph construction, and message passing grows rapidly in both time and memory, making some methods difficult to execute reliably under our hardware constraints. Therefore, for the large-scale dataset, we report the results of representative baseline methods that can be successfully executed under our computational environment, including Seurat, scGraph, sigGCN, scANVI, scGraphformer, GEDFN, and scRGCL. ScanNet maintains strong annotation performance on this large-scale dataset, achieving competitive or superior results compared with representative baseline methods across all evaluation metrics (Fig 2), indicating that ScanNet not only achieves strong performance across datasets of varying biological complexity, but also scales effectively to large single-cell atlases while maintaining robust annotation accuracy. Importantly, the performance advantage of ScanNet remains consistent across scales, suggesting that the incorporation of transcriptional regulatory priors provides stable benefits that are not diminished by increasing data size.

All these results demonstrate ScanNet achieves consistent classification advantages over tissues, species, and sample distribution. By systematically integrating TRN with gene expression data, ScanNet captures both regulatory mechanisms and transcriptional outcomes, providing mechanistic insights into cellular heterogeneity.

### ScanNet exhibits robust performance across different sequencing platforms

To evaluate ScanNet’s robustness to technical noise led by different sequencing platforms, we conduct experiments on PBMCbench dataset [37], which contains scRNA-seq data from the same donor’s peripheral blood mononuclear cells sequenced with seven different platforms.

**Fig 4:**
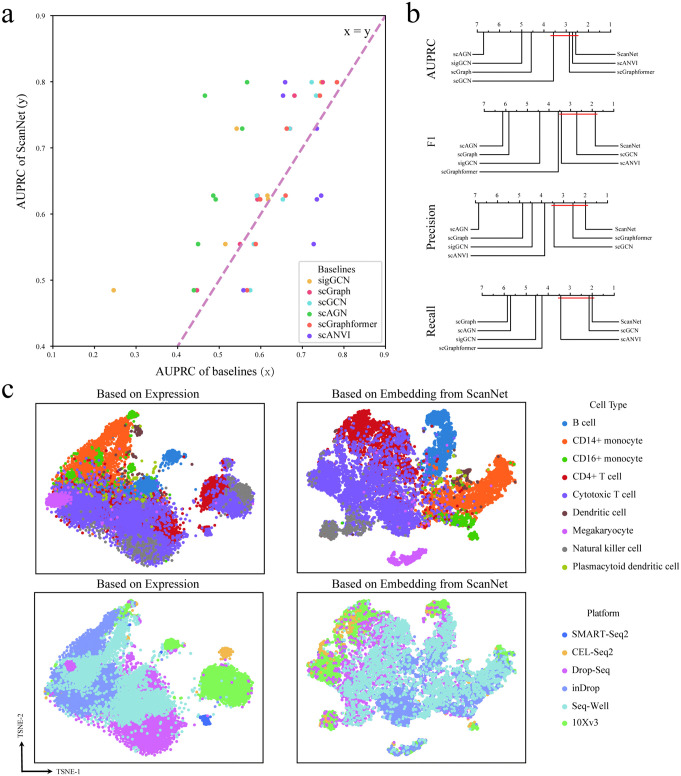
Evaluation of the generalization ability and robustness of ScanNet across different sequencing platforms. **(a)** Scatter plot comparing AUPRC scores of ScanNet with seven baselines across seven experimental settings. Each point corresponds to one comparison, with the x-axis representing baseline AUPRC and the y-axis representing ScanNet AUPRC. The dashed diagonal line (x = y) denotes performance parity. **(b)** Critical difference diagrams for four evaluation metrics (AUPRC, F1 score, Precision, and Recall). (c) t-SNE visualizations on the test set, colored by cell type (upper panels) and sequencing protocol (lower panels). The left panels show PCA-based t-SNE embeddings derived from the normalized and scaled expression profiles of selected TFs and TGs, while the right panels are based on embeddings learned by ScanNet, showing improved separation of cell types and reduced batch effects across protocols.

ScanNet is evaluated by leave-six-out validation, that is trained on data from one sequencing platform and tested on the remaining six, yielding a total of seven results. Fig 4a presents AUPRC pairwise comparisons between ScanNet and six baseline models. Most scatter points lie above the diagonal, demonstrating that ScanNet’s AUPRC outperforms that of most methods. In CD diagrams, ScanNet achieves the top overall ranking across all classification metrics, and exhibiting batch robustness comparable to that of the state-of-the-art method scANVI (Fig 4b).

Additionally, t-SNE visualizations compare cellular representations derived from the PCA-based embeddings of normalized expressions (Fig 4c, left panel) and ScanNet-generated embeddings (Fig 4c, right panel), with upper plots colored by cell type and lower plots by batch. In the expression-based visualization (left panel), different cell types show considerable overlap and mixing, making it difficult to distinguish clear cell-type boundaries. Moreover, when colored by sequencing platform (lower left), cells from the same platform tend to cluster together, indicating strong platform-driven batch effects. In contrast, the ScanNet embedding visualization (right panel) demonstrates markedly different patterns. When colored by cell type (upper right), ScanNet forms compact and well-separated clusters for each cell type, with clear boundaries between different cellular populations. Importantly, when colored by sequencing platform (lower right), ScanNet embeddings show reduced platform-driven clustering compared with the expression space, although some residual platform-associated structure remains in specific cell populations, such as cytotoxic T cells. The residual platform-specific structure is present to varying extents across methods, and that no method completely removes platform effects while simultaneously preserving clear cell-type separation (S4 Fig). Compared with other methods, ScanNet provides a more favorable balance between cell-type discrimination and cross-platform mixing. S3 Table summarizes the cross-platform classification metrics across seven experimental combinations and S4 Table records batch correction metrics of ScanNet and baselines.

By incorporating the underlying gene regulatory mechanisms and directed relationship from TFs to TGs, ScanNet captures the intrinsic regulatory processes that drive cellular identity, thereby mitigating platform-specific biases inherent to divergent sequencing technologies and exhibiting robust classification performance.

### ScanNet can identify novel cell types

ScanNet leverages prediction uncertainty quantified by entropy to distinguish novel cell types adapting to real-world scenarios. ScanNet exhibits higher uncertainty (higher entropy) when encountering previously unseen cell types compared to the confident predictions (lower entropy) for known cell types.

**Fig 5:**
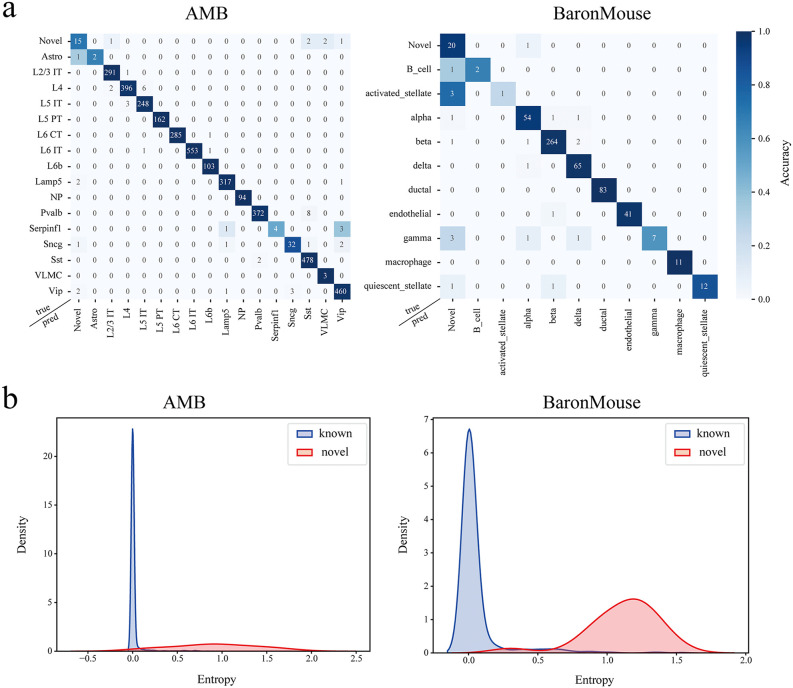
Identification of novel cell types by ScanNet. **(a)** Confusion matrices of ScanNet on the test set of AMB and BaronMouse datasets, showing that ScanNet can distinguish novel types from known cell types. **(b)** Entropy-based density distributions. The higher entropy values associated with novel cells demonstrate that prediction uncertainty can effectively separate unknown from known cell types.

In the experiment, cell types with fewer than 10 cells are removed from the training set and applied as entirely unknown novel cell types during testing. Unknown cell types (first row in each matrix) are predominantly classified as “Novel” rather than being assigned to known cell types (Fig 5a), indicating ScanNet demonstrates robust performance on both AMB and BaronMouse datasets, accurately predicting known cell types while effectively identifying novel cell types not encountered during training. Quantitatively, for the AMB dataset, ScanNet achieves 71.43% recall and 71.43% precision for novel cell detection. For the BaronMouse dataset, while precision is 66.67%, recall reaches an impressive 95.24%, highlighting ScanNet’s high sensitivity in identifying novel cells. In addition, we evaluate entropy-based novel cell-type identification for more cells, further demonstrating ScanNet can identify novel types with large number of cells (S4 Text and S5 Fig).

The entropy-based uncertainty quantification is effective and shows distinct distributions of entropy values in known and novel cell types (Fig 5b). In the AMB dataset, the mean and variance of known type distribution are 0.0168 and 0.0069, while these of novel type are 0.911 and 0.2155, implying that entropy of known types exhibit high prediction confidence. In contrast, novel cell types display broader distributions with higher entropy values, reflecting greater prediction uncertainty. This clear discrepancy validates entropy as an effective metric for distinguishing novel from known cell types based on model confidence.

These results demonstrate that ScanNet successfully identifies novel cell types through entropy-based uncertainty quantification, addressing a critical limitation of existing supervised models that misclassify unknown types into predefined categories. With high detection sensitivity, ScanNet provides practical functionality for real application scenarios where new cell populations continuously emerge, making it valuable for both exploratory research and clinical applications.

While ScanNet can identify cells that deviate from the reference distribution via entropy as “Novel”, such novel cells require further biological interpretation in practice. In real single-cell analysis pipelines, high-resolution annotation is typically achieved by combining automated predictions with marker-based validation. We therefore evaluate whether ScanNet can be integrated with marker-based tools to form a hybrid pipeline for improving the annotation of novel cells. Using scCATCH [51], a widely used marker-based tool, we adopt a stratified strategy in which ScanNet first partitions cells according to prediction confidence. High-confidence cells retain the labels predicted by ScanNet, whereas low-confidence cells are further annotated with scCATCH (S6 Fig). Importantly, for these low-confidence cells, scCATCH is applied to clusters derived from ScanNet embeddings rather than to clusters derived from the original expression profiles, corresponding results are provided in S7 Fig. This hybrid pipeline enhances the resolution of novel cell types. While ScanNet identifies such cells as “Novel”, scCATCH refines them into biologically meaningful candidate lineages, with reduced lineage confusion compared with standalone scCATCH based on the original expression profiles (S7 Fig). These results demonstrate that ScanNet can serve as an effective front-end module in practical annotation pipelines.

### ScanNet performs well with transferred scATAC-seq data

**Fig 6:**
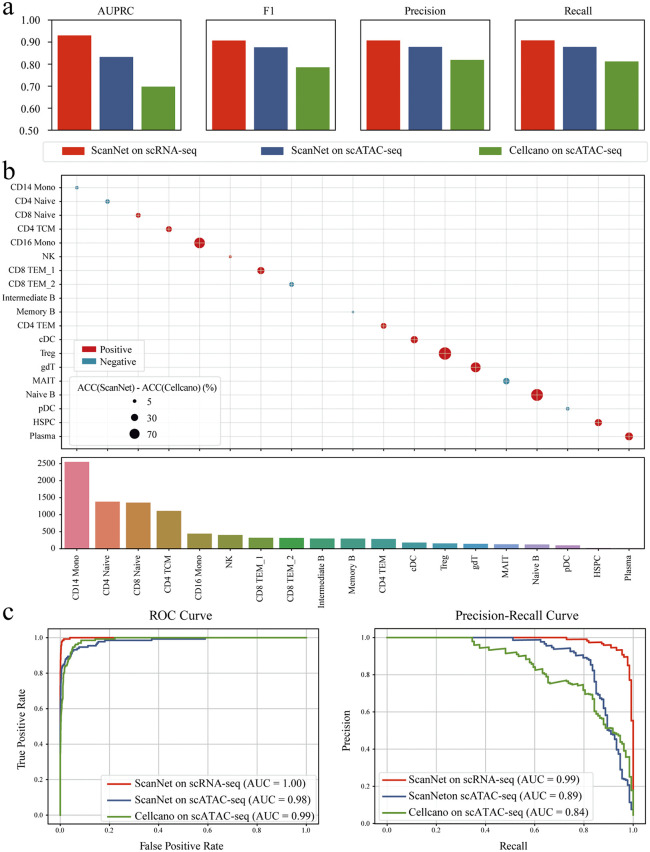
Annotation performance of ScanNet on scATAC-seq data. **(a)** Bar chart comparing annotation performance on the PBMC dataset. **(b)** Accuracy comparison between ScanNet and Cellcano across all cell types. The upper scatter plot depicts accuracy differences by cell type, where point size indicates the magnitude of the difference and color represents the performance direction (red: ScanNet superior; blue: Cellcano superior). The lower bar chart summarizes the number of cell types in the PBMC dataset. **(c)** The ROC and PRC of two models on the CD16 Mono type.

The framework of ScanNet can be transferred to other omics data, scATAC-seq data. To evaluate annotation performance on scATAC-seq data, annotation performance on scATAC-seq data is compared with Cellcano [41], a well-known cell classification tool specifically designed for scATAC-seq, implemented here with default hyperparameters. ScanNet is applied to paired scRNA-seq data and scATAC-seq data separately. ScanNet performs superior on scATAC-seq data than Cellcano across all evaluation metrics demonstrating the flexible extension ability of ScanNet from scRNA-Seq to scATAC-Seq data (Fig 6a).

We also evaluate ScanNet’s performance on sample size imbalance across types for scATAC-Seq data. Fig 6b presents a detailed accuracy comparison between ScanNet and Cellcano across all cell types in scATAC-seq data. The upper scatter plot visualizes accuracy differences by cell type where point size reflects the magnitude of differences and color denotes whose performance is better. Specifically, red represents ScanNet superior, blue stands Cellcano superior. The lower bar chart displays the sample size (number of cells) for each cell type shown in the scatter plot above, with both subplots sharing a common horizontal axis for cell-type alignment. ScanNet outperforms Cellcano in 12 out of 19 cell types, with particularly substantial advantages in minority cell types such as Naive B, gdT, and Treg cells, where ScanNet’s performance exceeds Cellcano by over 40–70%. In contrast, Cellcano shows marginal advantages only in 6 cell types, where the performance differences are relatively small (typically < 15%). However, Cellcano faces challenges when classifying cell types with limited sample sizes, often misclassifying them, as seen with CD16 Mono and Treg cells. In contrast, ScanNet demonstrates exceptional robustness to severe class imbalance, maintaining high performance across both abundant cell types and rare cell types (e.g., HSPC, Plasma with < 20 cells). ScanNet is also evaluated on scATAC-seq without paired scRNA-seq data and compared with Cellcano (S5 Table). ScanNet consistently outperforms Cellcano, indicating that ScanNet can generalize well to scATAC-seq datasets even when no matched scRNA-seq data are available, demonstrating its strong transferability and generalization across modalities.

To further exhibits the efficacy of ScanNet on minority cell types, we plot the receiver operating characteristic (ROC) and precision-recall curves (PRC) for the CD16 Mono population (Fig 6c). While ScanNet and Cellcano exhibit comparable AUROC performance (0.98 vs 0.99), ScanNet achieves a notable higher AUPRC (0.89 vs 0.84), highlighting its superior precision-recall trade-off in minority class annotation.

Although ScanNet’s performance on scATAC-seq data is slightly lower than its scRNA-seq performance, it still substantially outperforms the scATAC-seq-specific method Cellcano, demonstrating ScanNet’s effective cross-modal transferability and adaptability to diverse single-cell sequencing technologies.

## Discussions

Cell type annotation is a critical and fundamental task in single-cell omics research for serving as a critical bridge connecting fundamental mechanism exploration with clinical translation. However, the inherent feature of scRNA-seq data inevitably posed significant challenges to this task. To address these challenges, we propose ScanNet, an innovative model engineered to integrate reference TRN and scRNA-seq data via iterative heterogeneous graph convolution framework for cell annotation.

Benchmark experiment on eight benchmark datasets demonstrates ScanNet’s effectiveness in cell annotation tasks, where it outperforms existing methods across four metrics. Moreover, ScanNet exhibits strong recognition capabilities for minority cell types in imbalanced datasets. In batch generalization experiments, ScanNet effectively mitigates noise introduced by diverse sequencing platforms, as evidenced by t-SNE analysis showing that cell clustering is primarily driven by cell types rather than batch. To address the real application scenarios where unannotated datasets often contain previously unknown cell types, ScanNet accurately identifies novel classes in the simulated scenarios, demonstrating remarkable flexibility and generalization capabilities. Despite being originally designed for scRNA-seq data, ScanNet outperforms existing benchmark methods designed for scATAC-seq data, highlighting its versatility and robustness across diverse single-cell omics data. All these results indicate that ScanNet effectively integrates the causal relationships between transcriptional regulation and transcriptional expression, capturing cell heterogeneity through upstream regulatory mechanisms and achieving superior annotation performance.

In addition, we conduct ablation experiments of two embeddings and regulatory graph structure. The results confirm that ScanNet benefits from the complementary contributions of Expression-level and Regulation-level embeddings, and that the reference TRN contributes to classification accuracy (S8 Fig and S6 Table). We also further add a loss balancing and hyperparameter sensitivity analysis to examine the optimization dynamics of the MSE reconstruction loss and CE classification loss (S9 Fig), as well as the effects of different loss-weight settings (S7 Table) and L2 regularization coefficients (S8 Table). The results show that the two loss terms contribute complementarily during training and that ScanNet maintains stable performance across different weight ratios and regularization strengths. Besides, to investigate whether the learned attentions in ScanNet capture cell-type-specific regulatory differences, we reconstruct TF-TG interactions at the single-cell level (S5 Text). The reconstructed regulatory representations exhibit clear cell-type-specific organization (S10 Fig), which suggests that ScanNet has the potential to uncover cell-type-specific regulatory dependencies and provides a promising direction for future interpretability studies. We also compare ScanNet with ACTINN trained on all genes and find that ScanNet achieves better overall performance (S9 Table). Using more TFs and TGs yields no significant classification improvement (S10 Table). These results support using only TF and TG features in ScanNet.

ScanNet introduce the reference TRN network as priori biological knowledge, incorporating the regulatory logic and molecular mechanisms governing the gene expression, thereby enabling accurate cell annotation. ScanNet deploys a dual-channel encoder to dissect cellular heterogeneity by concurrently capturing transcriptional regulatory states and resultant gene expression. Specially, Regulation-level Encoder employs iterative heterogeneous node encoding optimization to capture the causal relationship between regulatory state information and expression outcomes by heterogeneous graph convolution, while the Expression-level Encoder extracts regulatory outcome features from scRNA-seq data within cells. This design allows for the extraction of fine-grained regulatory information at the gene level while simultaneously integrating broader contextual information at the cell level. Through dual-channel encoder, ScanNet constructs a unified and biologically informed cell representation that captures not only the overall transcriptional state but also the underlying regulatory mechanisms. The ablation results provide empirical evidence for the effectiveness of ScanNet’s dual-channel design and indicate that incorporating the TRN as prior information leads to improved performance.

Certainly, while ScanNet exhibits excellent performance in four annotation tasks, promising avenues for enhancement emerge from its design and experimental insights. First, a uniform TRN structure is applied to diverse cell types in ScanNet, without deeper exploration of cell-type-specific regulatory interactions that is critical for unraveling transcriptional divergence across biological contexts. Second, its capacity to resolve regulatory relationships and cell-type specificity could be strengthened by integrating multi-omics data, which would refine its ability to capture spatial and epigenetic determinants of cell identity. In future work, we aim to integrate finer-grained regulatory information, cell-type-specific regulatory relationship, with scRNA-seq and scATAC-seq data, thereby providing a more efficient, robust, and scalable solution for single-cell data analysis. This enhanced framework will not only dissect the causal regulatory circuits underlying cell identity specification but also empower researchers to collaboratively generate more valuable insights.

## Materials and method

### The dual-channel encoder

Transcriptional regulation orchestrates gene expression [26], establishing a causal link form TF to TG. With this prior knowledge, ScanNet deploys a dual-channel encoder, consists of a Regulation-level Encoder and an Expression-level Encoder, to dissect cellular heterogeneity from regulatory perspective and expression perspective respectively. The Regulation-level Encoder employs iHGCN to alternately update TF and TG representations through heterogeneous message passing on the TRN, combining self-projection, cross-projection, and attention-based fusion and learns cellular regulatory states, encoded into Regulation-level Embeddings. The Expression-level Encoder utilizes fully connected neural network to extract global cellular transcriptional states and regulatory outcome features inside cells from scRNA-seq data into Expression-level Embeddings, while neglecting inner regulation interactions within cells. The Regulation-level Encoder solely capture local features, due to learning filters’ localization property of GCN. Therefore, through the dual-channel encoder, ScanNet constructs a unified and biologically informed cell representation that captures not only the overall transcriptional state but also the underlying regulatory mechanisms. Comprehensive definitions of all variables, matrices, and operators used from data preprocessing through model training are provided in S11 Table.

### Iterative heterogeneous fusing encoding regulatory process in Regulation-level Encoder

iHGCN-based Regulation-level Encoder capture the regulatory dependencies by iteratively learning embeddings of TFs and TGs, which aggregates information from TGs to update TF representations, and vice versa. This bidirectional message passing enables Regulation-level Encoder to facilitate precise localization of cell states. The Regulation-level Encoder employs two iHGCN layers to integrate TF-TG regulatory relationships encoded in the TRN into the embeddings of TF and TG nodes, followed by a pooling layer to derive the Regulation-level Embedding for each cell. Taking the “Transcriptional Factor Update Step” in Fig 1b as an example, a detailed explanation of the iHGCN layer in Regulation-level Encoder is provided as follows. The iHGCN layer consists of two steps: heterogeneous feature extraction from TF-TG regulatory relationships, and attention-driven TF-TG representation fusion for cellular embedding generation.

### Heterogeneous feature extraction from TF-TG regulatory relationships

The iHGCN applies heterogeneous convolution to learn TF-TG regulatory associations from the TRN. In order to eliminate the influence of both TF and TG node degree on information transmission, row normalization is employed to the adjacency matrix, ensuring that the information from each node is appropriately represented during the aggregation.


$$\begin{array}{c} D^{T}=diag\left(\sum_{j=\text{1}}^{G}{|\tilde{A}}_{i,j}^{T-G}|\right)\in R^{T\times T}, \end{array}$$ (1)

$$\begin{array}{c} D^{G}=diag(\sum_{i=\text{1}}^{T}{|\tilde{A}}_{j,i}^{G-T}|)\in R^{G\times G}, \end{array}$$ (2)

$$\begin{array}{c} {\tilde{A}}_{n}^{T-G}={\left(D^{T}\right)}^{-\text{1}/\text{2}}{\tilde{A}}^{T-G}{\left(D^{G}\right)}^{-\text{1}/\text{2}}\;\in R^{T\times G}, \end{array}$$ (3)

$$\begin{array}{c} {\tilde{A}}_{n}^{G-T}={\left(D^{G}\right)}^{-\text{1}/\text{2}}{\tilde{A}}^{G-T}{\left(D^{T}\right)}^{-\text{1}/\text{2}}\;\in R^{G\times T}, \end{array}$$ (4)

where ${\tilde{A}}^{G-T}$ is the transpose of ${\tilde{A}}^{T-G}$, |·| denotes absolute values, and ∑ represents the row-wise sum. $D^{T}$ is the diagonal degree matrix of ${\tilde{A}}^{T-G}$, where each diagonal entry records the number of TGs regulated by the corresponding TF. Likewise, $D^{G}$ quantifies the number of TFs that regulate each TG.

The functions $f_{self-TF}$ and $f_{TF-TG}$ are designed to extract intrinsic features from TF nodes and regulatory information from TG nodes, respectively:


$$\begin{array}{c} \left\{ \begin{array}{c} @l{\tilde{H}}_{T-T}^{l}=f_{self-TF}(H_{T}^{l-\text{1}})=H_{T}^{l-\text{1}}W_{self-TF}^{l}\\ {\tilde{H}}_{T-G}^{l}=f_{TF-TG}(H_{G}^{l-\text{1}})={\tilde{A}}_{n}^{T-G}H_{G}^{l-\text{1}}W_{T-G}^{l} \end{array},\right. \end{array}$$ (5)

where $f_{self-TF}$ focuses on capturing the inherent characteristics of TFs, while $f_{TF-TG}$ uses graph convolution to integrate regulatory interaction information from neighboring TG nodes to infer TFs’ potential features. Specifically, $H_{T}^{l-\text{1}}\in R^{T\times d_{l-\text{1}}}$ and $H_{G}^{l-\text{1}}\in R^{G\times d_{l-\text{1}}}$ are the input feature of TF and TG of the $l^{th}$ layer (the output of ${\left(l-\text{1}\right)}^{th}$ layer), $W_{T-G}^{l}\in R^{d_{l-\text{1}}\times d_{l}}$ is the weight matrix of aggregating information from TG, $W_{self-TF}^{l}\in R^{d_{l-\text{1}}\times d_{l}}$ is the projection matrix to project $H_{T}^{l-\text{1}}$ into the same eigenspace as ${\tilde{H}}_{T-G}^{l}\in R^{T\times d_{l}}$.

However, the embedding ${\tilde{H}}_{T-G}^{l}$ based on TRN does not retain the feature information of the TF itself, potentially limiting the completeness of the feature representation. To overcome this limitation, iHGCN introduces an attention mechanism to integrate the embedding vector of TF itself (${\tilde{H}}_{T-T}^{l}$) and the inferred embedding vector of TF from TG (${\tilde{H}}_{T-G}^{l}$), thereby generating a more comprehensive and informative embedding for TF.

**Attention-driven TF-TG representation fusion and cellular embedding generation**: As depicted in Fig 1b, the attention mechanism maps a set of queries and a set of key-value pairs to an output. In practice, we pack together queries, keys and values into three matrixes Q, K and V respectively. Then attention mechanism can be formulated as: $softmax\left(f_{a}(Q,K)\right)V$, where $f_{a}$ is the attention function such as dot-product [52] or neural network [53]. Here, embedding vectors ${\tilde{H}}_{T-G}^{l}$ and ${\tilde{H}}_{T-T}^{l}$ are both as values matrix V. We define a weight matrix $W_{k}\in R^{d_{l}\times d_{a}}$ to map them into keys matrix K, and define a weight matrix $W_{q}\in R^{d_{l}\times d_{a}}$ to map ${\tilde{H}}_{T-T}^{l}$ into the query matrix Q, where $d_{a}$ is the hidden layer dimension. Formally,


$$\begin{array}{c} K_{T-T}={\tilde{H}}_{T-T}^{l}W_{k}, \end{array}$$ (6)

$$\begin{array}{c} K_{T-G}={\tilde{H}}_{T-G}^{l}W_{k}, \end{array}$$ (7)

$$\begin{array}{c} Q_{T}={\tilde{H}}_{T-T}^{l}W_{q}, \end{array}$$ (8)

Note that mapping ${\tilde{H}}_{T-T}^{l}$ as the query is the key to achieve personalized importance estimation for each TF node. The attention function is implemented as follows:


$$\begin{array}{c} e_{T-T}=ELU(\;[K_{T-T}∥Q_{T}]w_{a}^{T}), \end{array}$$ (9)

$$\begin{array}{c} e_{T-G}=ELU(\;[K_{T-G}∥Q_{T}]w_{a}^{T}), \end{array}$$ (10)

where ∥ denotes the row-wise concatenation operation, $w_{a}^{T}\in R^{\text{2}d_{a}\times \text{1}}$ is the parameter vector and ELU is the activation function. The $i^{th}$element of $e_{T-T}$ denotes the self-contribution weight of the $i^{th}$ TF, whereas the $i^{th}$ element of $e_{T-G}$ represents the regulation aggregated importance vector of its neighbors (TGs) on the $i^{th}$ TF. Then, the normalized attention coefficients are computed with the *softmax* function to update the representation of TF nodes:


$$\begin{array}{c} \;\left[{Att}_{T-T}∥{Att}_{T-G}\right]=softmax\left(\;\left[e_{T-T}∥e_{T-G}\right]\right), \end{array}$$ (11)

$$\begin{array}{c} H_{T}^{l}={Att}_{T-T}⊙{\tilde{H}}_{T-T}^{l}+{Att}_{T-G}⊙{\tilde{H}}_{T-G}^{l}, \end{array}$$ (12)

where $H_{T}^{l}$ is the $l^{th}$ iHGCN-updated TF embeddings, ${Att}_{T-T}$represents the normalized self-information attention weight for TFs, ${Att}_{T-G}$represents the normalized weight of regulatory information from TGs, and ⊙ is hadamard operator. The $l^{th}$ iHGCN-updatpled TG embeddings $H_{G}^{l}$ are obtained in an analogous manner.

The Regulation-level Encoder employs two iHGCN layers to learn molecular-level embeddings for TFs and TGs $\left(H_{T}^{\text{2}},{\;H}_{G}^{\text{2}}\right)$, and then aggregates them via a Read_Out function to produce cells’ Regulation-level Embeddings:


$$\begin{array}{c} H^{R}=Read\_Out([H_{T}^{\text{2}}∥H_{G}^{\text{2}}]), \end{array}$$ (13)

where Read_Out refers to the max-pooling layer, and $H^{R}\in R^{{N\times d}_{o}}$ represents the Regulation-level Embedding of cells, in which $d_{o}$ is the output dimension of the Regulation-level Encoder.

### Gene expression encoding in Expression-level Encoder

The Expression Encoder is designed to capture the holistic cellular state by modeling transcriptional profiles, thereby generating comprehensive global representations. This global perspective compensates for the limitation inherent to the Regulation Encoder, which focuses on gene-level regulatory relationship and consequently lacks a unified view of the entire cell system. Specifically, the Expression-level Encoder employs a simple MLP to capture the overall cellular state:


$$\begin{array}{c} H^{E}=Relu(Relu\left(XW_{\text{1}}+b_{\text{1}}\right)W_{\text{2}}+b_{\text{2}}), \end{array}$$ (14)

where $X=[X_{T}∥X_{G}]$ is the concatenated expression of TFs and TGs, representing gene expression within cells, $H^{E}\in R^{N\times d_{o}}$ is the Expression-level Embedding vector where $d_{o}$ is the output dimension of Expression-level Encoder same as that of Regulation-level Encoder, and Relu is the activation function.

### The decoder to reconstruct gene expression and the classifier to annotate cell type

The Reconstruction Decoder is designed to reconstruct downstream cell expressions from upstream Regulation-level Embeddings, ensuring that Regulation-level Embeddings accurately capture the regulatory state of cells. Thereby, the Reconstruction Decoder is designed to rebuild the expression of TFs and TGs from $H^{R}$:


$$\begin{array}{c} \hat{X}=[{\hat{X}}_{T}∥{\hat{X}}_{G}]=H^{R}W_{re}+b_{re}, \end{array}$$ (15)

where $W_{re}\in R^{d_{o}\times (T+G)}$ is the weight matrix, and $b_{re}\in R^{\left(T+G\right)}$ is the bias vector. As a result, $\hat{X}\in R^{N\times (T+G)}$ has the same dimension as the concatenated input $X=\;[X_{T}∥X_{G}]$, which T+G is the number of all TF nodes and TG nodes in TRN.

It is worth noting that the reconstruction objective is applied only to the gene expression matrix, rather than the TRN. In our framework, the TRN is incorporated as a shared prior across all cells to guide information propagation between transcription factors and target genes. Since this prior is identical for all cells, introducing a reconstruction loss on the TRN would implicitly enforce the model to recover the same graph structure across samples. However, the primary objective of cell type annotation is to capture cell-specific transcriptional variations. Therefore, constraining the model to reconstruct a global and invariant network structure may reduce its ability to learn discriminative representations. For this reason, we restrict the reconstruction objective to the gene expression matrix, which directly reflects cell-level heterogeneity, while the TRN is used as structural prior knowledge rather than a reconstruction target.

Since Regulation-level Embeddings $H^{R}$ and Expression-level Embeddings $H^{E}$ characterize cell features from regulatory mechanisms and expression outcomes, the Classifier is trained on the concatenation of these two embeddings to achieve accurate cell annotation:


$$\begin{array}{c} z=\;[H^{R}∥H^{E}]W_{cl}+b_{cl}, \end{array}$$ (16)

$$\begin{array}{c} \hat{Y}=softmax(z), \end{array}$$ (17)

where $W_{cl}\in R^{\text{2}d_{o}\times C}$ is the weight matrix in which C is the number of cell types, $b_{cl}\in R^{C}$ is the bias vector, and $\hat{Y}\in R^{N\times C}$ is the predicted probabilities matrix.

### The optimization of loss function

The loss function comprises three key components: the reconstruction loss of expression data $L_{MSE}$, the classification loss of cell typing $L_{CE}$ and L2 regularization, as given in Eq (18)–(20) respectively,


$$\begin{array}{c} L_{MSE}=\frac{\text{1}}{N}\sum_{i=\text{1}}^{N}{\left(X_{i}-{\hat{X}}_{i}\right)}^{\text{2}}, \end{array}$$ (18)

$$\begin{array}{c} L_{CE}=-\frac{\text{1}}{N}\sum_{i=\text{1}}^{N}\sum_{j=\text{1}}^{C}Y_{ij}\text{log}({\hat{Y}}_{ij}), \end{array}$$ (19)

$$\begin{array}{c} L=L_{MSE}+L_{CE}+\lambda \sum w^{\text{2}}, \end{array}$$ (20)

$L_{MSE}$ is the reconstruction loss between normalized $X=\;[X_{T}∥X_{G}]$ and reconstructed expression data $\hat{X}=[{\hat{X}}_{T}∥{\hat{X}}_{G}]$ to minimize the loss of regulatory information that occurs when converting embeddings from gene to cell within Regulation-level Encoder; $L_{CE}$ is the classification loss of ground truth labels Y and predicted probabilities $\hat{Y}$ to enhance classification performance; and $\lambda \sum w^{\text{2}}$ is L2 regularization applied to all parameters ensuring a more stable training process and improved robustness. By minimizing L with the *SGD* optimizer, ScanNet preserves gene regulatory information to the greatest extent while simultaneously enhancing cell type prediction through the inferred cell potential embedding.

### Recognize novel types by prediction uncertainty

In real application scenarios, the emergence of previous unknown cell types, also referred as novel types, is frequently encountered. In other words, real cell data not only contain known types trained during training process but may also include novel cell types. Therefore, prediction uncertainty is introduced as a quantitative metric of output confidence, while keeping the model architecture unchanged. Prediction uncertainty is quantified via the entropy of the predicted probability distribution derived from classifier outputs, where higher entropy indicates a more uniform distribution, reflecting greater uncertainty in predictions, and lower entropy implies greater confidence.

To emulate real application scenarios, the experimental design is as follows:


$$\begin{array}{c} X_{test}^{\prime }=X_{test}\cup X_{novel}, \end{array}$$ (21)

$$\begin{array}{c} {\hat{Y}}_{test}=ScanNet(X_{test}^{\prime },A^{T-G}), \end{array}$$ (22)

where $X_{novel}$ represents cells of novel types, not encountered in training, and is integrated into the test set $X_{test}$ to create a new test set $X_{test}^{\prime }$ containing known and novel cell types, then $X_{test}^{\prime }$ is fed into the pre-trained ScanNet to obtain the probability distribution ${\hat{Y}}_{test}$ over predefined classes. We hypothesize that prediction uncertainty, quantified via entropy, will be higher for cells of novel types compared to known types. Hence, prediction uncertainty is calculated for each cell and cells with high uncertainty are identified as novel types. Mathematically, given the probability distribution ${\hat{Y}}_{test}$ from the Classifier, the prediction uncertainty is calculated as Eq (23):


$$\begin{array}{c} E_{i}=-\sum_{j=\text{1}}^{C}{\hat{Y}}_{ij}\text{log}({\hat{Y}}_{ij}), \end{array}$$ (23)

where ${\hat{Y}}_{ij}$ denotes the predicted probability that the $i^{th}$ cell belongs to the $j^{th}$ class in ${\hat{Y}}_{test}$, C represents the number of predefined classes during training and $E_{i}$ is the prediction uncertainty of $i^{th}$ cell.

Then, the decision is made by comparing prediction uncertainty $E_{i}$ with the threshold t:


$$\begin{array}{c} {\hat{y}}_{i}=\left\{ \begin{array}{c} @cC_{novel}\;if\;E_{i}>t\\ {argmax}_{j}\left({\hat{Y}}_{ij}\right)\;if\;E_{i}\leq t \end{array}\right.\;\;\;\;, \end{array}$$ (24)

When the prediction uncertainty exceeds t, the probability distribution is more dispersed, reflecting less confidence on the prediction, and such cells are assigned as novel $C_{novel}$. Conversely, if the prediction uncertainty is less than t, ScanNet show high confidence on the prediction and the cell is assigned to the class with the highest probability. In this manuscript, the default value of threshold t is set to 0.75. $C_{novel}$ indicates that the cell belongs to novel types not encountered during prior training.

### Code availability

The code used for model training and evaluation is available on GitHub (https://github.com/Ying-Lab/ScanNet).

## Supporting information

S1 Table Description of the PBMCbench dataset. (DOCX)

S2 Table Detailed performance metrics for cell annotation comparison. (DOCX)

S3 Table Cross-platform performance comparison. (DOCX)

S4 Table Batch correction comparison. (DOCX)

S5 Table Annotation performance on scATAC-seq data. (DOCX)

S6 Table Ablation analysis of embedding and graph in ScanNet. (DOCX)

S7 Table ScanNet performance under different loss weighting schemes. (DOCX)

S8 Table ScanNet performance under different L2 regularization coefficient. (DOCX)

S9 Table Performance comparison between ScanNet and ACTINN (all genes). (DOCX)

S10 Table Robustness of ScanNet to variations in TF and TG sizes. (DOCX)

S11 Table Description of variables and operators. (DOCX)

S1 Fig Schematic diagram of transferring ScanNet to scATAC-seq data. (DOCX)

S2 Fig Critical difference diagrams for F1 and Recall. (DOCX)

S3 Fig Quantity statistics of the PBMC dataset. (DOCX)

S4 Fig T-SNE visualizations on the test set of all baselines. (DOCX)

S5 Fig Accuracy confusion matrix in detecting novel types for more cells. (DOCX)

S6 Fig Integration of ScanNet with marker gene-based annotation workflow. (DOCX)

S7 Fig Annotation of novel type cells using the integrated ScanNet-scCATCH workflow and scCATCH alone. (DOCX)

S8 Fig Boxplots of embedding and graph ablation in ScanNet. (DOCX)

S9 Fig Training dynamics of MSE and CE losses on the Muraro dataset. (DOCX)

S10 Fig Clustering of cell types based on reconstructed TRN. (DOCX)

S1 Text Data preprocessing. (DOCX)

S2 Text Experimental design of ScanNet transferred to scATAC-seq. (DOCX)

S3 Text Performance metrics. (DOCX)

S4 Text ScanNet can identify novel types for more cells. (DOCX)

S5 Text Reconstruction of cell-specific TF-TG regulatory networks. (DOCX)
