# Supplementary material for: ScanNet: Single-cell annotation informed by transcriptional regulation Network via iterative heterogeneous graph learning
Source: PLoS Comput Biol. 2026 Aug 6;22(8):e1014602. doi: 10.1371/journal.pcbi.1014602 (PMC13446671; doi:10.1371/journal.pcbi.1014602)
Supplement: Supplementary file 1 [file pcbi.1014602.s001.docx]

**S1 Table. Description of the PBMCbench dataset.**

| **Dataset** | **Source** | **Sequencing Platforms** | **Specie** | **TF** | **TG** | **Cell** | **Cell Type** |
| --- | --- | --- | --- | --- | --- | --- | --- |
| PBMCbench | Peripheral blood mononuclear cell | 10X Chromium v2 | Homo | 92 | 794 | 6444 | 9 |
|  |  | 10X Chromium v3 |  |  |  | 3222 | 8 |
|  |  | CEL-Seq2 |  |  |  | 253 | 7 |
|  |  | Drop-Seq |  |  |  | 3222 | 9 |
|  |  | SMART-Seq2 |  |  |  | 253 | 6 |
|  |  | Seq-Well |  |  |  | 3176 | 7 |
|  |  | inDrop |  |  |  | 3222 | 7 |
