# Supplementary material for: ScanNet: Single-cell annotation informed by transcriptional regulation Network via iterative heterogeneous graph learning
Source: PLoS Comput Biol. 2026 Aug 6;22(8):e1014602. doi: 10.1371/journal.pcbi.1014602 (PMC13446671; doi:10.1371/journal.pcbi.1014602)
Supplement: Supplementary file 2 [file pcbi.1014602.s002.docx]

**S2 Table. Detailed performance metrics for cell annotation comparison.**

|  | **PBMC** | **Muraro** | **BMMC** | **AMB** | **TM** | **Baron**  **Mouse** | **Chen**  **Mouse** | **Immune** |
| --- | --- | --- | --- | --- | --- | --- | --- | --- |
|  | **AUPRC** | | | | | | | |
| **scAGN** | 0.7089 | 0.6192 | 0.6528 | 0.3581 | 0.1870 | 0.4718 | 0.3608 | - |
| **scGCN** | 0.8935 | 0.9566 | 0.7816 | 0.5358 | 0.5574 | 0.7249 | 0.4982 | - |
| **sigGCN** | 0.7468 | 0.9716 | 0.8260 | 0.9412 | 0.9498 | 0.9469 | 0.2824 | 0.9128 |
| **Seurat** | 0.8908 | 0.9278 | 0.7726 | 0.9549 | 0.9219 | 0.9355 | 0.5170 | 0.8815 |
| **ACTINN** | 0.9118 | 0.9433 | 0.8221 | 0.9674 | 0.9325 | 0.8807 | 0.5247 | - |
| **scGraph** | 0.9031 | 0.9619 | 0.6433 | 0.9729 | 0.7068 | 0.9449 | 0.2994 | 0.7502 |
| **GEDFN** | 0.8750 | 0.9749 | 0.8138 | 0.9160 | 0.9044 | 0.8701 | 0.6006 | 0.9097 |
| **scANVI** | 0.8905 | 0.9805 | 0.8278 | 0.9560 | 0.9578 | 0.9508 | 0.5857 | 0.9236 |
| **scGraph-former** | 0.9047 | 0.9607 | 0.7591 | 0.8964 | 0.5615 | 0.9716 | **0.6029** | 0.6678 |
| **scRGCL** | 0.9119 | 0.9516 | 0.8131 | 0.9571 | 0.9296 | 0.9605 | 0.5238 | 0.9296 |
| **ScanNet** | **0.9299** | **0.9869** | **0.8393** | **0.9907** | **0.9612** | **0.9920** | 0.5934 | **0.9388** |
|  | **F1** | | | | | | | |
| **scAGN** | 0.7396 | 0.3517 | 0.6308 | 0.2353 | 0.2377 | 0.4829 | 0.3879 | - |
| **scGCN** | 0.8576 | 0.8751 | 0.8117 | 0.2586 | 0.7562 | 0.8775 | 0.5253 | - |
| **sigGCN** | 0.7961 | 0.9672 | 0.8319 | 0.9867 | 0.9662 | 0.9627 | 0.3769 | 0.8878 |
| **Seurat** | 0.8816 | 0.9671 | 0.8134 | 0.9789 | 0.9444 | 0.9339 | 0.4816 | 0.8458 |
| **ACTINN** | 0.8962 | 0.9512 | 0.8328 | 0.9790 | 0.9605 | 0.9294 | 0.5370 | - |
| **scGraph** | 0.8645 | 0.9581 | 0.7313 | 0.9811 | 0.8614 | 0.9351 | 0.3127 | 0.7923 |
| **GEDFN** | 0.8853 | 0.9672 | 0.8219 | 0.9763 | 0.9591 | 0.9057 | 0.5372 | 0.8867 |
| **scANVI** | 0.8804 | 0.9627 | 0.8321 | 0.9863 | 0.9697 | 0.9547 | 0.5876 | 0.8914 |
| **scGraph-former** | 0.8465 | 0.9687 | 0.7912 | 0.9666 | 0.8430 | 0.9565 | 0.5747 | 0.8158 |
| **scRGCL** | 0.7794 | 0.9448 | 0.8086 | 0.9723 | 0.9496 | 0.4873 | 0.2144 | 0.8919 |
| **ScanNet** | **0.9070** | **0.9718** | **0.8366** | **0.9897** | **0.9730** | **0.9762** | **0.5987** | **0.8957** |
|  | **Precision** | | | | | | | |
| **scAGN** | 0.7145 | 0.2794 | 0.6251 | 0.1744 | 0.1697 | 0.3943 | 0.3774 | - |
| **scGCN** | 0.8891 | 0.8523 | 0.8146 | 0.4254 | 0.7356 | 0.8600 | 0.5423 | - |
| **sigGCN** | 0.7869 | 0.9681 | 0.8331 | 0.9861 | 0.9667 | 0.9662 | 0.3442 | 0.8906 |
| **Seurat** | 0.8899 | 0.9679 | 0.8113 | 0.9783 | 0.9466 | 0.9274 | 0.5283 | 0.8525 |
| **ACTINN** | 0.8959 | 0.9523 | 0.8332 | 0.9799 | 0.9617 | 0.9225 | 0.5396 | - |
| **scGraph** | 0.8731 | 0.9603 | 0.7237 | 0.9832 | 0.8487 | 0.9412 | 0.4659 | 0.7717 |
| **GEDFN** | 0.8854 | 0.9682 | 0.8210 | 0.9744 | 0.9592 | 0.8950 | 0.5953 | 0.8874 |
| **scANVI** | 0.8765 | 0.9641 | 0.8332 | 0.9853 | 0.9706 | 0.9540 | 0.5870 | 0.8917 |
| **scGraphformer** | 0.8691 | 0.9694 | 0.7853 | 0.9686 | 0.8247 | 0.9622 | 0.5946 | 0.8004 |
| **scRGCL** | 0.8520 | 0.9493 | 0.8322 | 0.9723 | 0.9542 | 0.7087 | 0.3748 | 0.8941 |
| **ScanNet** | **0.9742** | **0.9726** | **0.8368** | **0.9899** | **0.9735** | **0.9776** | **0.6238** | **0.8957** |
|  | **Recall** | | | | | | | |
| **scAGN** | 0.7910 | 0.4984 | 0.6888 | 0.3902 | 0.4064 | 0.6250 | 0.4810 | - |
| **scGCN** | 0.8761 | 0.9009 | 0.8220 | 0.4014 | 0.8027 | 0.8982 | 0.5669 | - |
| **sigGCN** | 0.8111 | 0.9670 | 0.8360 | 0.9878 | 0.9670 | 0.9661 | 0.4657 | 0.8886 |
| **Seurat** | 0.8882 | 0.9670 | 0.8194 | 0.9800 | 0.9463 | 0.9446 | 0.5299 | 0.8495 |
| **ACTINN** | 0.8986 | 0.9513 | 0.8350 | 0.9797 | 0.9618 | 0.9375 | 0.5466 | - |
| **scGraph** | 0.8637 | 0.9575 | 0.7488 | 0.9802 | 0.8826 | 0.9321 | 0.3330 | 0.8193 |
| **GEDFN** | 0.8862 | 0.9670 | 0.8246 | 0.9784 | 0.9606 | 0.9196 | 0.5076 | 0.8876 |
| **scANVI** | 0.8969 | 0.9623 | 0.8362 | 0.9873 | 0.9704 | 0.9625 | **0.6003** | 0.8926 |
| **scGraphformer** | 0.8512 | 0.9686 | 0.8120 | 0.9696 | 0.8762 | 0.9607 | 0.5970 | 0.8381 |
| **scRGCL** | 0.8076 | 0.9434 | 0.8197 | 0.9735 | 0.9516 | 0.4554 | 0.2568 | 0.8924 |
| **ScanNet** | **0.9076** | **0.9717** | **0.8382** | **0.9899** | **0.9735** | **0.9768** | 0.5869 | **0.8958** |

*Note: Bold represents the optimal value of corresponding dataset.*
