# Supplementary material for: ScanNet: Single-cell annotation informed by transcriptional regulation Network via iterative heterogeneous graph learning
Source: PLoS Comput Biol. 2026 Aug 6;22(8):e1014602. doi: 10.1371/journal.pcbi.1014602 (PMC13446671; doi:10.1371/journal.pcbi.1014602)
Supplement: Supplementary file 3 [file pcbi.1014602.s003.docx]

**S3 Table.** **Cross-platform performance comparison.**

| **Train set** | **Model** | **AUPRC** | **F1** | **Precision** | **Recall** |
| --- | --- | --- | --- | --- | --- |
| **10Xv2** | **scAGN** | 0.5671 | 0.7449 | 0.7304 | 0.7757 |
|  | **scGCN** | 0.7223 | 0.7949 | 0.788 | 0.8139 |
|  | **sigGCN** | 0.7457 | 0.7883 | 0.8066 | 0.7812 |
|  | **scGraph** | 0.7489 | 0.7318 | 0.7942 | 0.715 |
|  | **scANVI** | 0.6586 | 0.7632 | 0.787 | 0.7656 |
|  | **scGraphformer** | 0.7832 | 0.7798 | 0.8226 | 0.7755 |
|  | **ScanNet** | 0.7993 | 0.8102 | 0.8228 | 0.8069 |
| **10Xv3** | **scAGN** | 0.5554 | 0.7284 | 0.7086 | 0.7590 |
|  | **scGCN** | 0.6696 | 0.7678 | 0.7645 | 0.7852 |
|  | **sigGCN** | 0.5420 | 0.6432 | 0.6833 | 0.6361 |
|  | **scGraph** | 0.6702 | 0.6995 | 0.7884 | 0.6618 |
|  | **scANVI** | 0.7349 | 0.6862 | 0.7488 | 0.6749 |
|  | **scGraphformer** | 0.6624 | 0.7389 | 0.7757 | 0.7555 |
|  | **ScanNet** | 0.7291 | 0.7983 | 0.8029 | 0.8012 |
| **CEL-Seq2** | **scAGN** | 0.4391 | 0.4222 | 0.3662 | 0.557 |
|  | **scGCN** | 0.5747 | 0.7683 | 0.7658 | 0.7815 |
|  | **sigGCN** | 0.2461 | 0.3832 | 0.4438 | 0.3755 |
|  | **scGraph** | 0.4465 | 0.3918 | 0.6402 | 0.3952 |
|  | **scANVI** | 0.5583 | 0.5784 | 0.6196 | 0.5760 |
|  | **scGraphformer** | 0.5671 | 0.6364 | 0.7334 | 0.6381 |
|  | **ScanNet** | 0.4847 | 0.6696 | 0.704 | 0.669 |
| **Drop-Seq** | **scAGN** | 0.4656 | 0.5397 | 0.5458 | 0.5927 |
|  | **scGCN** | 0.7332 | 0.7072 | 0.7744 | 0.7054 |
|  | **sigGCN** | 0.7409 | 0.6782 | 0.7703 | 0.6654 |
|  | **scGraph** | 0.6810 | 0.6335 | 0.7133 | 0.6115 |
|  | **scANVI** | 0.6536 | 0.6970 | 0.7721 | 0.6783 |
|  | **scGraphformer** | 0.7422 | 0.5990 | 0.7814 | 0.5951 |
|  | **ScanNet** | 0.779 | 0.6841 | 0.7808 | 0.6738 |
| **SMART-Seq2** | **scAGN** | 0.4492 | 0.3993 | 0.3497 | 0.5269 |
|  | **scGCN** | 0.5833 | 0.7155 | 0.7130 | 0.7482 |
|  | **sigGCN** | 0.5148 | 0.7216 | 0.7156 | 0.7381 |
|  | **scGraph** | 0.5501 | 0.6241 | 0.6692 | 0.6362 |
|  | **scANVI** | 0.7273 | 0.6544 | 0.7107 | 0.6619 |
|  | **scGraphformer** | 0.5879 | 0.7441 | 0.7386 | 0.7715 |
|  | **ScanNet** | 0.5545 | 0.7354 | 0.7287 | 0.7581 |
| **Seq-Well** | **scAGN** | 0.4857 | 0.3953 | 0.3647 | 0.5252 |
|  | **scGCN** | 0.5904 | 0.6899 | 0.7087 | 0.7345 |
|  | **sigGCN** | 0.6160 | 0.6946 | 0.6830 | 0.7292 |
|  | **scGraph** | 0.5918 | 0.6858 | 0.6771 | 0.7059 |
|  | **scANVI** | 0.7453 | 0.8020 | 0.8112 | 0.8071 |
|  | **scGraphformer** | 0.6597 | 0.6981 | 0.6780 | 0.678 |
|  | **ScanNet** | 0.6278 | 0.7102 | 0.6913 | 0.743 |
| **inDrop** | **scAGN** | 0.4916 | 0.4429 | 0.3913 | 0.5652 |
|  | **scGCN** | 0.6530 | 0.7519 | 0.7301 | 0.7885 |
|  | **sigGCN** | 0.6177 | 0.7633 | 0.7446 | 0.7889 |
|  | **scGraph** | 0.592 | 0.6810 | 0.6730 | 0.7007 |
|  | **scANVI** | 0.7349 | 0.8347 | 0.8407 | 0.8461 |
|  | **scGraphformer** | 0.5986 | 0.7502 | 0.7376 | 0.7787 |
|  | **ScanNet** | 0.6221 | 0.7642 | 0.7463 | 0.7909 |
