# Supplementary material for: ScanNet: Single-cell annotation informed by transcriptional regulation Network via iterative heterogeneous graph learning
Source: PLoS Comput Biol. 2026 Aug 6;22(8):e1014602. doi: 10.1371/journal.pcbi.1014602 (PMC13446671; doi:10.1371/journal.pcbi.1014602)
Supplement: Supplementary file 4 [file pcbi.1014602.s004.docx]

**S4 Table. Batch correction comparison.**

| **Metric** | **ScanNet** | **scGraph-former** | **scGCN** | **scGraph** | **sigGCN** | **scAGN** | **scANVI** |
| --- | --- | --- | --- | --- | --- | --- | --- |
| **BRAS** | 0.96 | 0.97 | 0.91 | 1.00 | 1.00 | 0.71 | 0.39 |
| **Graph**  **Connectivity** | 1.00 | 0.97 | 0.92 | 0.89 | 0.93 | 0.77 | 0.71 |
