# Supplementary material for: ScanNet: Single-cell annotation informed by transcriptional regulation Network via iterative heterogeneous graph learning
Source: PLoS Comput Biol. 2026 Aug 6;22(8):e1014602. doi: 10.1371/journal.pcbi.1014602 (PMC13446671; doi:10.1371/journal.pcbi.1014602)
Supplement: Supplementary file 5 [file pcbi.1014602.s005.docx]

**S5 Table. Annotation performance on scATAC-seq data.**

| **Model** | **AUPRC** | **F1** | **Precision** | **Recall** |
| --- | --- | --- | --- | --- |
| **Paired scATAC-seq** | | | | |
| **ScanNet** | 0.8324 | 0.8762 | 0.8780 | 0.8779 |
| **Cellcano** | 0.6974 | 0.7853 | 0.8191 | 0.8121 |
| **Unpaired scATAC-seq** | | | | |
| **ScanNet** | 0.8704 | 0.8887 | 0.8888 | 0.8896 |
| **Cellcano** | 0.7209 | 0.8279 | 0.8382 | 0.8481 |
