# Supplementary material for: ScanNet: Single-cell annotation informed by transcriptional regulation Network via iterative heterogeneous graph learning
Source: PLoS Comput Biol. 2026 Aug 6;22(8):e1014602. doi: 10.1371/journal.pcbi.1014602 (PMC13446671; doi:10.1371/journal.pcbi.1014602)
Supplement: Supplementary file 6 [file pcbi.1014602.s006.docx]

**S6 Table. Ablation analysis of embedding and graph in ScanNet.**

| **Dataset** | **ScanNet** | **Only Expression Embedding** | **Only Regulation Embedding** | **Randomized**  **Graph** |
| --- | --- | --- | --- | --- |
| **AUPRC** | | | | |
| **PBMC** | 0.9299 | 0.8719 | 0.8444 | 0.7768 |
| **Muraro** | 0.9869 | 0.9680 | 0.9429 | 0.5456 |
| **BMMC** | 0.8393 | 0.8292 | 0.8193 | 0.7859 |
| **AMB** | 0.9907 | 0.9035 | 0.9382 | 0.8217 |
| **TM** | 0.9612 | 0.9391 | 0.9199 | 0.8601 |
| **BaronMouse** | 0.9920 | 0.9438 | 0.8140 | 0.6007 |
| **ChenMouse** | 0.5934 | 0.5808 | 0.5029 | 0.4452 |
| **F1** | | | | |
| **PBMC** | 0.907 | 0.8901 | 0.8731 | 0.7831 |
| **Muraro** | 0.9718 | 0.9685 | 0.9103 | 0.5095 |
| **BMMC** | 0.8366 | 0.8354 | 0.8203 | 0.7782 |
| **AMB** | 0.9897 | 0.9752 | 0.9760 | 0.9402 |
| **TM** | 0.973 | 0.9663 | 0.9586 | 0.936 |
| **BaronMouse** | 0.9762 | 0.9563 | 0.8999 | 0.8249 |
| **ChenMouse** | 0.5987 | 0.5934 | 0.5537 | 0.5039 |
| **Precision** | | | | |
| **PBMC** | 0.9075 | 0.8921 | 0.8777 | 0.8124 |
| **Muraro** | 0.9726 | 0.9692 | 0.915 | 0.6253 |
| **BMMC** | 0.8368 | 0.8346 | 0.8201 | 0.7825 |
| **AMB** | 0.9899 | 0.9740 | 0.9754 | 0.9395 |
| **TM** | 0.9735 | 0.9665 | 0.9593 | 0.9365 |
| **BaronMouse** | 0.9776 | 0.9605 | 0.8853 | 0.8155 |
| **ChenMouse** | 0.6238 | 0.5939 | 0.5632 | 0.5092 |
| **Recall** | | | | |
| **PBMC** | 0.9076 | 0.892 | 0.8768 | 0.7955 |
| **Muraro** | 0.9717 | 0.9686 | 0.9135 | 0.5047 |
| **BMMC** | 0.8382 | 0.8367 | 0.822 | 0.7789 |
| **AMB** | 0.9899 | 0.9766 | 0.9768 | 0.9425 |
| **TM** | 0.9735 | 0.9668 | 0.9598 | 0.9379 |
| **BaronMouse** | 0.9768 | 0.9607 | 0.9196 | 0.8554 |
| **ChenMouse** | 0.5869 | 0.6206 | 0.5764 | 0.5414 |
