# Supplementary material for: ScanNet: Single-cell annotation informed by transcriptional regulation Network via iterative heterogeneous graph learning
Source: PLoS Comput Biol. 2026 Aug 6;22(8):e1014602. doi: 10.1371/journal.pcbi.1014602 (PMC13446671; doi:10.1371/journal.pcbi.1014602)
Supplement: Supplementary file 7 [file pcbi.1014602.s007.docx]

**S7 Table. ScanNet Performance under Different Loss Weighting Schemes.**

| MSE:CE | PBMC | Muraro | BMMC | AMB | TM | Baron  Mouse | Chen  Mouse |
| --- | --- | --- | --- | --- | --- | --- | --- |
| 1:2 | 0.8960 | 0.9614 | 0.8307 | 0.9691 | 0.9547 | 0.9781 | 0.5735 |
| 2:1 | 0.9053 | 0.9784 | 0.8321 | 0.9684 | 0.9531 | 0.9869 | 0.5739 |
| 1:1 | **0.9299** | **0.9869** | **0.8393** | **0.9907** | **0.9612** | **0.9920** | **0.5934** |

*Note: Bold represents the optimal value of corresponding dataset.*
