# Supplementary material for: ScanNet: Single-cell annotation informed by transcriptional regulation Network via iterative heterogeneous graph learning
Source: PLoS Comput Biol. 2026 Aug 6;22(8):e1014602. doi: 10.1371/journal.pcbi.1014602 (PMC13446671; doi:10.1371/journal.pcbi.1014602)
Supplement: Supplementary file 8 [file pcbi.1014602.s008.docx]

**S8 Table. ScanNet Performance under Different L2 Regularization coefficient.**

| L2 regularization coefficient | PBMC | Muraro | BMMC | AMB | TM | Baron  Mouse | Chen  Mouse |
| --- | --- | --- | --- | --- | --- | --- | --- |
| $1\times{10}^{-5}$ | 0.9239 | 0.9696 | 0.8291 | 0.9841 | 0.9515 | 0.9872 | 0.5765 |
| $1\times{10}^{-3}$ | 0.9171 | 0.9798 | 0.8385 | 0.9843 | 0.9595 | 0.9808 | 0.5792 |
| $1\times{10}^{-4}$ | **0.9299** | **0.9869** | **0.8393** | **0.9907** | **0.9612** | **0.9920** | **0.5934** |

*Note: Bold represents the optimal value of corresponding dataset.*
