# Supplementary material for: ScanNet: Single-cell annotation informed by transcriptional regulation Network via iterative heterogeneous graph learning
Source: PLoS Comput Biol. 2026 Aug 6;22(8):e1014602. doi: 10.1371/journal.pcbi.1014602 (PMC13446671; doi:10.1371/journal.pcbi.1014602)
Supplement: Supplementary file 9 [file pcbi.1014602.s009.docx]

**S9 Table. Performance comparison between ScanNet and ACTINN (all genes).**

|  | **AUPRC** | **F1** | **Precision** | **Recall** |
| --- | --- | --- | --- | --- |
| **Muraro** | | | | |
| **ScanNet** | 0.9869 | 0.9718 | 0.9726 | 0.9717 |
| **ACTINN*** | 0.9719 | 0.9686 | 0.9692 | 0.9686 |
| **AMB** | | | | |
| **ScanNet** | 0.9907 | 0.9897 | 0.9899 | 0.9899 |
| **ACTINN*** | 0.9840 | 0.9896 | 0.9901 | 0.9901 |

*Note: * denotes results obtained with ACTINN trained on all genes.*
