# Supplementary material for: ScanNet: Single-cell annotation informed by transcriptional regulation Network via iterative heterogeneous graph learning
Source: PLoS Comput Biol. 2026 Aug 6;22(8):e1014602. doi: 10.1371/journal.pcbi.1014602 (PMC13446671; doi:10.1371/journal.pcbi.1014602)
Supplement: Supplementary file 10 [file pcbi.1014602.s010.docx]

**S10 Table. Robustness of ScanNet to variations in TF and TG sizes.**

| **Dataset** | $\mathbf{TF}^{\boldsymbol{a}}$ | $\mathbf{TG}^{\boldsymbol{a}}$ | $\mathbf{AURPC}^{\boldsymbol{a}}$ | $\mathbf{TF}^{\boldsymbol{b}}$ | $\mathbf{TG}^{\boldsymbol{b}}$ | $\mathbf{AURPC}^{\boldsymbol{b}}$ |
| --- | --- | --- | --- | --- | --- | --- |
| **PBMC** | 266 | 684 | 0.9299 | 869 | 5418 | 0.9241 |
| **Muraro** | 106 | 798 | 0.9869 | 518 | 3005 | 0.9839 |
| **BMMC** | 213 | 673 | 0.8356 | 568 | 3443 | 0.8332 |
| **AMB** | 52 | 362 | 0.9907 | 246 | 1208 | 0.9863 |
| **TM** | 90 | 979 | 0.9612 | 349 | 1959 | 0.9708 |
| **BaronMouse** | 112 | 1006 | 0.9920 | 654 | 4044 | 0.9916 |
| **ChenMouse** | 245 | 657 | 0.5934 | 302 | 1414 | 0.0492 |

*Note:*$a$ *denotes preprocessing with highly variable gene (HVG) filtering, while* $b$ *denotes preprocessing without HVG filtering.*
