# Supplementary material for: ScanNet: Single-cell annotation informed by transcriptional regulation Network via iterative heterogeneous graph learning
Source: PLoS Comput Biol. 2026 Aug 6;22(8):e1014602. doi: 10.1371/journal.pcbi.1014602 (PMC13446671; doi:10.1371/journal.pcbi.1014602)
Supplement: Supplementary file 11 [file pcbi.1014602.s011.docx]

# S11 Table. Description of variables and operators.

| **Variable** | **Description** |
| --- | --- |
| $\boldsymbol{N}$ | the number of cells |
| $\boldsymbol{M}$ | the number of all genes |
| $\boldsymbol{H}$ | the number of HVGs |
| $\boldsymbol{T}$ | the number of dataset-specific TFs |
| $\boldsymbol{G}$ | the number of dataset-specific TGs |
| $\boldsymbol{d}_{\boldsymbol{a}}$ | the attention hidden dimension |
| $\boldsymbol{d}_{\boldsymbol{o}}$ | the output dimension of Regulation-level Encoder and Expression-level Encoder |
| $\boldsymbol{C}$ | the number of cell types |
| **Matrix** | **Description** |
| $\boldsymbol{A}^{\boldsymbol{T-G}}$ | the original adjacency matrix of the reference TRN, where rows correspond to TFs and columns correspond to TGs |
| $\tilde{\boldsymbol{X}}$ | the normalized HVG expression matrix |
| $\boldsymbol{X}_{\boldsymbol{T}}$ | the normalized and dataset-specific TF expression matrix, columns correspond to TF retained from the reference TRN |
| $\boldsymbol{X}_{\boldsymbol{G}}$ | the normalized and dataset-specific TG expression matrix, columns correspond to TG retained from the reference TRN |
| ${\tilde{\boldsymbol{A}}}^{\boldsymbol{T-G}}$**/**${\tilde{\boldsymbol{A}}}^{\boldsymbol{G}\mathbf{-}\boldsymbol{T}}$ | the dataset-specific adjacency matrix of TRN, where rows correspond to retained TF/TG and columns correspond to retained TG/TF |
| $\boldsymbol{D}^{\boldsymbol{T}}$**/**$\boldsymbol{D}^{\boldsymbol{G}}$ | the diagonal degree matrix of $\tilde{A}^{T-G}$/$\tilde{A}^{G-T}$, where each diagonal entry records the number of TGs regulated by the corresponding TF/the number of TFs regulating the corresponding TG |
| ${\tilde{\boldsymbol{A}}}_{n}^{T-G}$**/**${\tilde{\boldsymbol{A}}}_{n}^{G-T}$ | the standardized adjacency matrix of TRN |
| $\boldsymbol{H}_{\boldsymbol{T}}^{\boldsymbol{l}\mathbf{-1}}$**/**$\boldsymbol{H}_{\boldsymbol{G}}^{\boldsymbol{l}\mathbf{-1}}$ | the input feature of TF/TG in the $l^{th}$ iHGCN layer, as well as the output of TF/TG in the ${(l-1)}^{th}$ iHGCN layer |
| ${\tilde{\boldsymbol{H}}}_{\boldsymbol{T-T}}^{\boldsymbol{l}}$ | intrinsic features derived from the TF itself in the $l^{th}$ iHGCN layer |
| ${\tilde{\boldsymbol{H}}}_{\boldsymbol{T}\mathbf{-}\boldsymbol{G}}^{\boldsymbol{l}}$ | TF-related features inferred from neighboring TGs based on the reference TRN in the $l^{th}$ iHGCN layer |
| $\boldsymbol{e}_{\boldsymbol{T-T}}$ | the unnormalized self-contribution importance vector, where the $i^{th}$ element represents the weight of the $i^{th}$ TF |
| $\boldsymbol{e}_{\boldsymbol{T}\mathbf{-}\boldsymbol{G}}$ | the unnormalized cross-contribution importance vector, where the $i^{th}$ element represents the influence of TGs on the $i^{th}$ TF |
| $\boldsymbol{Att}_{\boldsymbol{T-T}}$**/**$\boldsymbol{Att}_{\boldsymbol{T}\mathbf{-}\boldsymbol{G}}$ | the normalized importance weight from TFs/TGs to TFs |
| $\boldsymbol{H}^{\boldsymbol{R}}$ | the regulation-level embedding |
| $\boldsymbol{H}^{\boldsymbol{E}}$ | the expression-level embedding |
| $\hat{\boldsymbol{X}}$ | the reconstructed expression matrix of TFs and TGs |
| $\hat{\boldsymbol{Y}}$ | the predicted probabilities matrix |
| $\boldsymbol{Y}$ | the true cell-type label |
| $\boldsymbol{X}_{\boldsymbol{test}}$ | the normalized expression matrix of the test set |
| $\boldsymbol{X}_{\boldsymbol{novel}}$ | the normalized expression matrix of novel types |
| $\boldsymbol{X}_{\boldsymbol{test}}^{\mathbf{'}}$ | the union of $X_{test}$ and $X_{novel}$ |
| $\boldsymbol{E}_{\boldsymbol{i}}$ | the prediction uncertainty for $i^{th}$ cell |
| ${\hat{\boldsymbol{y}}}_{\boldsymbol{i}}$ | the predicted type of the $i^{th}$ cell |
| **Operator** | **Description** |
| $\left\vert\boldsymbol{\cdot} \right\vert$ | absolute values |
| $\boldsymbol{\sum}$ | the row-wise sum |
| $\boldsymbol{diag}$ | a diagonalization operator that maps a vector to a square diagonal matrix whose diagonal entries correspond to the vector components |
| $\boldsymbol{\parallel}$ | the row-wise concatenation |
| $\boldsymbol{\odot}$ | Hadamard operator, row-wise broadcasting multiplication |
