# Supplementary material for: ScanNet: Single-cell annotation informed by transcriptional regulation Network via iterative heterogeneous graph learning
Source: PLoS Comput Biol. 2026 Aug 6;22(8):e1014602. doi: 10.1371/journal.pcbi.1014602 (PMC13446671; doi:10.1371/journal.pcbi.1014602)
Supplement: Supplementary file 12 [file pcbi.1014602.s012.docx]

**S1 Fig. Schematic diagram of transferring ScanNet to scATAC-seq data.**

**S1 Fig. Schematic diagram of transferring ScanNet to scATAC-seq data.** The scATAC-seq peak accessibility matrix is converted into a gene score matrix using Signac, from which TF and TG score matrices are derived. The TF and TG score matrices, together with the reference TRN, are provided as inputs to ScanNet.
