# Supplementary material for: ScanNet: Single-cell annotation informed by transcriptional regulation Network via iterative heterogeneous graph learning
Source: PLoS Comput Biol. 2026 Aug 6;22(8):e1014602. doi: 10.1371/journal.pcbi.1014602 (PMC13446671; doi:10.1371/journal.pcbi.1014602)
Supplement: Supplementary file 13 [file pcbi.1014602.s013.docx]

**S2 Fig. Critical difference diagrams for F1 and Recall.**

**S2 Fig. Critical difference diagrams for F1 and Recall.** Methods connected by red are not significantly different in performance according to the Nemenyi test.
