# Supplementary material for: ScanNet: Single-cell annotation informed by transcriptional regulation Network via iterative heterogeneous graph learning
Source: PLoS Comput Biol. 2026 Aug 6;22(8):e1014602. doi: 10.1371/journal.pcbi.1014602 (PMC13446671; doi:10.1371/journal.pcbi.1014602)
Supplement: Supplementary file 14 [file pcbi.1014602.s014.docx]

**S3 Fig. Quantity statistics of the PBMC dataset.**

**S3 Fig. Quantity statistics of the PBMC dataset.** Bar plot showing the number of samples assigned to each cell type (x-axis), with counts reported on the y-axis. Cell types are ordered by decreasing number.
