# Supplementary material for: ScanNet: Single-cell annotation informed by transcriptional regulation Network via iterative heterogeneous graph learning
Source: PLoS Comput Biol. 2026 Aug 6;22(8):e1014602. doi: 10.1371/journal.pcbi.1014602 (PMC13446671; doi:10.1371/journal.pcbi.1014602)
Supplement: Supplementary file 15 [file pcbi.1014602.s015.docx]

**S4 Fig.** **T-SNE visualizations on the test set of all baselines.**

**S4 Fig. T-SNE visualizations on the test set of all baselines.** Residual platform-specific structure is present to varying extents across methods.
