# Supplementary material for: ScanNet: Single-cell annotation informed by transcriptional regulation Network via iterative heterogeneous graph learning
Source: PLoS Comput Biol. 2026 Aug 6;22(8):e1014602. doi: 10.1371/journal.pcbi.1014602 (PMC13446671; doi:10.1371/journal.pcbi.1014602)
Supplement: Supplementary file 16 [file pcbi.1014602.s016.docx]

**S5 Fig. Accuracy confusion matrix** **in detecting novel types for more cells.**

**S5 Fig. Accuracy confusion matrix in detecting novel types for more cells.** Confusion matrices summarizing performance on the AMB (left) and BaronMouse (right) datasets when evaluating novel-type detection with a larger number of cells. The color indicates per-class accuracy and numbers indicate the corresponding counts.
