# Supplementary material for: ScanNet: Single-cell annotation informed by transcriptional regulation Network via iterative heterogeneous graph learning
Source: PLoS Comput Biol. 2026 Aug 6;22(8):e1014602. doi: 10.1371/journal.pcbi.1014602 (PMC13446671; doi:10.1371/journal.pcbi.1014602)
Supplement: Supplementary file 17 [file pcbi.1014602.s017.docx]

**S6 Fig. Integration of ScanNet with marker gene-based annotation workflow.**

**S6 Fig. Integration of ScanNet with marker gene-based annotation workflow**. Schematic overview of the integrated annotation pipeline. ScanNet first generates cell-level predictions, probability distributions, and low-dimensional embeddings. Based on prediction entropy, cells are partitioned into high-confidence (low entropy) and low-confidence (high entropy) groups. High-confidence cells retain their predicted labels, while low-confidence cellsare further clustered in the ScanNet embedding space and annotated using the marker gene–based method scCATCH. The final annotation is obtained by combining ScanNet predictions with scCATCH-derived labels for low-confidence cells.
