# Supplementary material for: ScanNet: Single-cell annotation informed by transcriptional regulation Network via iterative heterogeneous graph learning
Source: PLoS Comput Biol. 2026 Aug 6;22(8):e1014602. doi: 10.1371/journal.pcbi.1014602 (PMC13446671; doi:10.1371/journal.pcbi.1014602)
Supplement: Supplementary file 18 [file pcbi.1014602.s018.docx]

**S7 Fig. Annotation of novel type cells using the integrated ScanNet-scCATCH workflow and scCATCH alone.**

**S7 Fig. Annotation of novel type cells using the integrated ScanNet-scCATCH workflow and scCATCH alone**. Sankey diagram showing the flow of novel cells through the integrated annotation process (a) and scCATCH alone (b). Most novel cells are identified as low-confidence by ScanNet and subsequently assigned to biologically meaningful lineages by scCATCH, such as T cells and mesenchymal stem cells. The refined labels show improved alignment with true cell identities, demonstrating that the integrated workflow effectively translates statistically “Novel” cells into biologically interpretable categories. Compared to the integrated workflow, scCATCH alone exhibits substantial lineage confusion, with immune-related cells misclassified as B cells and schwann cells inconsistently mapped to pericytes or mesenchymal stem cells. These results highlight the limitations of standalone marker-based annotation and the benefit of incorporating ScanNet-derived representations.
