# Supplementary material for: ScanNet: Single-cell annotation informed by transcriptional regulation Network via iterative heterogeneous graph learning
Source: PLoS Comput Biol. 2026 Aug 6;22(8):e1014602. doi: 10.1371/journal.pcbi.1014602 (PMC13446671; doi:10.1371/journal.pcbi.1014602)
Supplement: Supplementary file 19 [file pcbi.1014602.s019.docx]

**S8 Fig. Boxplots of embedding and graph ablation in ScanNet.**

**S8 Fig. Boxplots of embedding and graph ablation in ScanNet.** Boxplots summarize the annotation performance across seven benchmark scRNA-seq datasets under different ablation settings. Orange and blue correspond to ScanNet using only the Expression-level Embedding and only the Regulation-level Embedding, respectively. Green denotes the Regulation-only setting where the TRN is replaced by a randomized graph. The full ScanNet model achieves the best overall performance, indicating that expression-derived global transcriptional features and regulation-derived TF–TG features provide complementary information for cell-type annotation. To further assess whether the regulatory graph itself contributes biologically meaningful information, the reference TRN was replaced with a randomized graph of matched sparsity under the Regulation-only setting. The performance decline observed after graph randomization indicates that the Regulation-level Embedding benefits from the specific regulatory relationships encoded in the TRN.
