# Supplementary material for: ScanNet: Single-cell annotation informed by transcriptional regulation Network via iterative heterogeneous graph learning
Source: PLoS Comput Biol. 2026 Aug 6;22(8):e1014602. doi: 10.1371/journal.pcbi.1014602 (PMC13446671; doi:10.1371/journal.pcbi.1014602)
Supplement: Supplementary file 20 [file pcbi.1014602.s020.docx]

**S9 Fig. Training dynamics of MSE and CE losses on the Muraro dataset.**

**S9 Fig. Training dynamics of MSE and CE losses on the Muraro dataset.** (a) Loss curves of the mean squared error (MSE) loss and cross-entropy (CE) loss over training epochs. The MSE loss exhibits a larger numerical scale throughout training, while both losses rapidly decrease and converge. (b) Corresponding gradient magnitudes of the two loss terms, measured by the L2 norm of gradients with respect to all model parameters. The reported values are averaged over all samples within each epoch.
