# Supplementary material for: ScanNet: Single-cell annotation informed by transcriptional regulation Network via iterative heterogeneous graph learning
Source: PLoS Comput Biol. 2026 Aug 6;22(8):e1014602. doi: 10.1371/journal.pcbi.1014602 (PMC13446671; doi:10.1371/journal.pcbi.1014602)
Supplement: Supplementary file 21 [file pcbi.1014602.s021.docx]

**S10 Fig. Clustering of cell types based on reconstructed TRN.**

**S10 Fig.** **Clustering of cell types based on reconstructed TRN.** t-SNE visualization of cell-specific reconstructed TF–TG regulatory matrices inferred by ScanNet in the AMB and BaronMouse datasets. Each point represents a single cell, and colors indicate annotated cell types. (a) In the AMB dataset, reconstructed regulatory representations show clear cell-type-dependent organization, with major neuronal and non-neuronal populations forming distinct or locally structured clusters. (b) In the BaronMouse dataset, non-endocrine populations such as ductal, endothelial, macrophage, and quiescent stellate cells are clearly separated from endocrine populations, while endocrine cell types show partial proximity consistent with their related regulatory programs. These results suggest that the reconstructed TF-TG regulatory representations capture cell-type-associated regulatory heterogeneity.
