# Supplementary material for: ScanNet: Single-cell annotation informed by transcriptional regulation Network via iterative heterogeneous graph learning
Source: PLoS Comput Biol. 2026 Aug 6;22(8):e1014602. doi: 10.1371/journal.pcbi.1014602 (PMC13446671; doi:10.1371/journal.pcbi.1014602)
Supplement: Supplementary file 22 [file pcbi.1014602.s022.docx]

# S1 Text. Data preprocessing.

**Construction of heterogeneous graph base on Reference TRN**: In this paper, we use the archived transcription factor network from CollectTRI [1] as reference TRN. CollectTRI collects 43,175 signed TF-TG regulations (activation or repression) for 1,184 TFs from multiple cell types and tissues, covering both human and mouse data.

As the TRN is composed of two biologically distinct types of regulatory elements, TFs and TGs, with uni-directional edges from TFs to TGs, thus constituting a heterogeneous graph. This structural constraint renders conventional graph embedding algorithms inadequate, as they assume bidirectional node connectivity patterns that are inherently absent in TRNs.

Therefore, reference TRN is modeled as a heterogeneous graph $\mathcal{G=}\left( \nu^{T},\nu^{G},\boldsymbol{A}^{T-G} \right)$, where $\nu^{T}$ indicates the set of transcript factors, $\nu^{G}$ indicates the set of target genes, and $A^{T-G}\in R^{|\nu^{T}|\times|\nu^{G}|}$ is the adjacency matrix depicting regulatory connection. The elements in matrix $\boldsymbol{A}^{T-G}$ are defined as Eq (1).

$\boldsymbol{A}_{i,j}^{T-G}=\left\{ \begin{aligned} 1 activation regulation \\ -1 repression regulation \\ 0 no regulation \end{aligned} \right.$,	(1)

To ensure the graph in each experiment retains sufficient yet non-redundant information, we construct dataset-specific heterogeneous graphs based on the reference TRN. For each dataset, only those TF-TG relationships that are present in that specific dataset are retained from the complete TRN, resulting in a heterogeneous subgraph tailored to each dataset. Consequently, the number of regulatory interactions in the input graph varies across different datasets, with each dataset's graph representing a strict subgraph of the original TRN.

**Normalized Gene Expression Profile:** scRNA-seq data measures gene expression profiles for cells, denoted as a matrix $\boldsymbol{X}\in R^{N\times M}$, where $N$ is the number of cells, $M$ is the number of genes and each element $X_{ij}$ indicates the read counts of the $j^{th}$ gene in the $i^{th}$ cell. Logarithmic Normalization [2] is performed to normalize the counts in each cell as shown in Eq (2),

$\tilde{X}_{ij}=log(1+s\cdot\frac{X_{ij}}{\sum_{m} X_{im}})$,	(2)

where$m$ is the gene index $(m=1, 2, 3\cdots M)$ and $s$ is the scaling value set as ${10}^{4}$. To select highly variable genes (HVGs) for capturing cellular heterogeneity and reducing data dimension, we calculate the variance of each gene across all cells and select the top-$H$ genes [3] ($H$ = 3000 in our work) to get the HVG expression matrix $\tilde{\boldsymbol{X}}\in R^{N\times H}$. A dataset-specific TRN is constructed by extracting an induced subnetwork from the reference TRN based on the selected HVGs. Specifically, $\tilde{\boldsymbol{X}}$ is intersected with $\left( \nu^{T},\nu^{G} \right)$ in $\mathcal{G}$ to obtain TF expression data $\boldsymbol{X}_{T}\in R^{N\times T}( T \leq|\nu^{T}|)$ and expression of target genes $\boldsymbol{X}_{G}\in R^{N\times G}(G\leq|\nu^{G}|)$, where $T$ and $G$ are the numbers of retained TFs and TGs. As a result, the dataset-specific subgraph $\tilde{\mathcal{G}}$ is extracted from the reference graph $\mathcal{G}$ with adjacency matrix ${\tilde{\boldsymbol{A}}}^{T-G}\in R^{T\times G}$.

1.	Müller-Dott S, Tsirvouli E, Vazquez M, Ramirez Flores RO, Badia-i-Mompel P, Fallegger R, et al. Expanding the coverage of regulons from high-confidence prior knowledge for accurate estimation of transcription factor activities. Nucleic Acids Research. 2023;51(20):10934-49.

2.	Yang R, Dai W, Li C, Zou J, Wu D, Xiong H. scBiGNN: Bilevel Graph Representation Learning for Cell Type Classification from Single-cell RNA Sequencing Data. arXiv preprint arXiv:231210310. 2023.

3.	Wang T, Bai J, Nabavi S. Single-cell classification using graph convolutional networks. BMC Bioinformatics. 2021;22(1):364.
