# Supplementary material for: ScanNet: Single-cell annotation informed by transcriptional regulation Network via iterative heterogeneous graph learning
Source: PLoS Comput Biol. 2026 Aug 6;22(8):e1014602. doi: 10.1371/journal.pcbi.1014602 (PMC13446671; doi:10.1371/journal.pcbi.1014602)
Supplement: Supplementary file 23 [file pcbi.1014602.s023.docx]

**S2 Text. Experimental design of ScanNet transferred to scATAC-seq.**

To generate a gene score matrix compatible with ScanNet, we process the scATAC-seq data using Signac [1], a computational framework specifically designed for scATAC-seq analysis. Signac facilitates the efficient transformation of raw chromatin accessibility data into quantifiable representations of gene score. Notably, all data in the PBMC dataset are uniformly mapped to the hg38 genome reference sequence. The detailed experimental design is outlined as follows.

We generate a gene score matrix by Signac, denoted as $\boldsymbol{S}$, where each element represents the calculated activity of a gene based on chromatin accessibility. To ensure consistency across modalities, we filter genes in $\boldsymbol{S}$ based on scRNA-seq from matched tissue (the RNA-seq data from the PBMC dataset in our work). Specifically, TFs and TGs retained in the RNA-seq-preprocessed expression matrices $\boldsymbol{X}_{T}$ and $\boldsymbol{X}_{G}$ are used to filter the gene score matrix $\boldsymbol{S}$, yielding the ScanNet-compatible input matrices $\boldsymbol{S}_{T}$ and $\boldsymbol{S}_{G}$. For genes absent in $\boldsymbol{S}$, we assign a minimal activity value of $1\times{10}^{-5}$ to maintain shape compatibility between $\boldsymbol{X}_{T}$ and $\boldsymbol{S}_{T}$, as well as $\boldsymbol{X}_{G}$ and $\boldsymbol{S}_{G}$ in shape. For scATAC-seq data without paired scRNA-seq, the gene score matrix can be processed using the same preprocessing pipeline as scRNA-seq data to select TFs and TGs, allowing ScanNet to operate on scATAC-seq data in a standalone manner. Then $\boldsymbol{S}_{T}$ and $\boldsymbol{S}_{G}$ are fed into ScanNet for training:

$\hat{Y}=ScanNet(\boldsymbol{S}_{T},\boldsymbol{S}_{G},\boldsymbol{A}^{T-G})$,	(1)

1.	Aibar S, González-Blas CB, Moerman T, Huynh-Thu VA, Imrichova H, Hulselmans G, et al. SCENIC: single-cell regulatory network inference and clustering. Nature Methods. 2017;14(11):1083-6.
