# Supplementary material for: ScanNet: Single-cell annotation informed by transcriptional regulation Network via iterative heterogeneous graph learning
Source: PLoS Comput Biol. 2026 Aug 6;22(8):e1014602. doi: 10.1371/journal.pcbi.1014602 (PMC13446671; doi:10.1371/journal.pcbi.1014602)
Supplement: Supplementary file 24 [file pcbi.1014602.s024.docx]

**S3 Text.** **Performance metrics.**

When evaluate the performance of ScanNet and baseline methods, Weighted-F1, Weighted-Precision, Weighted-Recall and Area Under Precision-Recall Curve (AUPRC) are used.

$Weighted-Precision=\sum_{i=1}^{C} \frac{n_{i}}{N}P_{i}, where P_{i}=\frac{{TP}_{i}}{{TP}_{i}+{FP}_{i}}$,	(1)

$Weighted-Recall=\sum_{i=1}^{C} \frac{n_{i}}{N}R_{i}, where R_{i}=\frac{{TP}_{i}}{{TP}_{i}+{FN}_{i}}$,	(2)

$Weighted-F1=\sum_{i=1}^{C} \frac{n_{i}}{N}{F1}_{i}, where {F1}_{i}=\frac{2*P_{i}*R_{i}}{P_{i}+R_{i}}$,	(3)

Where ${TP}_{i}$, ${FP}_{i}$, and ${FN}_{i}$ denote the number of samples belonging to category $i$ that are correctly classified, the number of samples from other categories misclassified as category $i$, and the number of samples from category $i$ misclassified as other categories, respectively. And $n_{i}$ denotes the number of true samples in the $i^{th}$ class and $N$ represents the total number of samples across all categories. While AUPRC is calculated as the area under the Precision-Recall curve.
