# Supplementary material for: ScanNet: Single-cell annotation informed by transcriptional regulation Network via iterative heterogeneous graph learning
Source: PLoS Comput Biol. 2026 Aug 6;22(8):e1014602. doi: 10.1371/journal.pcbi.1014602 (PMC13446671; doi:10.1371/journal.pcbi.1014602)
Supplement: Supplementary file 25 [file pcbi.1014602.s025.docx]

**S4 Text. ScanNet can identify novel types for more cells.**

To validate the applicability of entropy quantification to more cells, we conduct additional experiments in which cell types with larger cell numbers are selected as novel types, rather than restricting the analysis to rare cell populations. Specifically, we designated the Sncg cell type (123 cells) as novel in the AMB dataset and the endothelial cell type (139 cells) as novel in the BaronMouse dataset. ScanNet was trained on the remaining cell types (excluding the designated novel type) and then evaluated on a test set that includes the novel type.
