# Supplementary material for: ScanNet: Single-cell annotation informed by transcriptional regulation Network via iterative heterogeneous graph learning
Source: PLoS Comput Biol. 2026 Aug 6;22(8):e1014602. doi: 10.1371/journal.pcbi.1014602 (PMC13446671; doi:10.1371/journal.pcbi.1014602)
Supplement: Supplementary file 26 [file pcbi.1014602.s026.docx]

# S5 Text. Reconstruction of cell-specific TF-TG regulatory networks.

In ScanNet, the TRN is represented as a heterogeneous graph consisting of TF nodes and TG nodes. The Regulation-level Encoder performs iterative heterogeneous graph convolution between these two node types and learns node-level attention coefficients to adaptively balance the intrinsic node representation and the regulatory information aggregated from its cross-type neighbors. However, these attention coefficients are defined at the node level rather than directly on individual TF-TG edges. Therefore, to derive cell-specific regulatory interactions, we decomposed the node-level attention weights into edge-level contributions based on the linearity of the neighborhood aggregation operator. Fig 1 illustrates the flow of TRN reconstruction with reverse decomposition.

**Fig 1. The flow of TRN reconstruction with reverse decomposition**.

Let $\alpha^{self} and \alpha^{neigh}$ denote the normalized attention weight $\boldsymbol{Att}_{T-T}$ and $\boldsymbol{Att}_{T-G}$ of TF nodes in the manuscript. And let $\beta^{self} and \beta^{neigh}$ denote the normalized attention weight $\boldsymbol{Att}_{G-G}$ and $\boldsymbol{Att}_{G-T}$ of TG nodes.

**Message passing from TGs to TFs**

For the $i^{th}$ TF, the regulatory signal aggregated from its connected TG neighbors can be written as:

$$\begin{aligned} {\tilde{\boldsymbol{h}}}_{i}^{\text{T}-\text{G}}=\sum_{j\in\mathcal{N}\left( i \right)} w_{ij}^{T-G}\cdot\boldsymbol{h}_{j}^{G},\#\left( 1 \right) \end{aligned}$$

where $\mathcal{N}\left( i \right)$ denotes the set of TG neighbors connected to $i^{th}$ TF and $w_{ij}^{T-G}$ is the element of matrix ${\tilde{\boldsymbol{A}}}_{n}^{T-G}$. The updated TF representation is obtained by combining the intrinsic TF representation and the aggregated TG-derived regulatory signal:

$$\begin{aligned} \boldsymbol{h}_{i}^{T}=\alpha_{i}^{self}\cdot{\tilde{\boldsymbol{h}}}_{i}^{T-T}+\alpha_{i}^{neigh}\cdot{\tilde{\boldsymbol{h}}}_{i}^{\text{T}-\text{G}}, \end{aligned}\left( 2 \right)$$

where $\alpha_{i}^{self}$ and $\alpha_{i}^{neigh}$are node-level attention coefficients for $i^{th}$ TF, representing the relative contributions of its intrinsic information and TG-neighborhood-derived regulatory information, respectively.

Substituting the aggregation term into the update equation gives:

$$\boldsymbol{h}_{i}^{\text{T}}=\alpha_{i}^{self}\cdot{\tilde{\boldsymbol{h}}}_{i}^{\text{T}-\text{T}}+\alpha_{i}^{neigh}\cdot\sum_{j\in\mathcal{N}\left( i \right)} w_{ij}^{\text{T}-\text{G}}\cdot{\tilde{\boldsymbol{h}}}_{j}^{G}$$

$$\begin{aligned} =\alpha_{i}^{self}\cdot{\tilde{\boldsymbol{h}}}_{i}^{\text{T}-\text{T}}+\sum_{j\in\mathcal{N}\left( i \right)} \left( \alpha_{i}^{neigh}\cdot w_{ij}^{T-G} \right)\cdot{\tilde{\boldsymbol{h}}}_{j}^{G},\#\left( 3 \right) \end{aligned}$$

Thus, from the TF-side update, the effective contribution of the edge between $i^{th}$ TF and $j^{th}$ TG can be defined as:

$$\begin{aligned} \tilde{w}_{ij}^{\text{T}-\text{G}}=\alpha_{i}^{neigh}\cdot w_{ij}^{T-G},\#\left( 4 \right) \end{aligned}$$

This term measures how strongly the $i^{th}$ TF depends on the regulatory information propagated from its TG neighborhood in a specific cell, projected onto the TF-TG edge ($i, j$).

**Message passing from TFs to TGs**

Similarly, for the $j^{th}$ TG, the upstream regulatory signal aggregated from connected TFs is defined as:

$$\begin{aligned} {\tilde{\boldsymbol{h}}}_{j}^{G-T}=\sum_{i\in\mathcal{N}\left( j \right)} w_{ji}^{G-T}\cdot\boldsymbol{h}_{i}^{T},\#\left( 5 \right) \end{aligned}$$

where $\mathcal{N}\left( j \right)$ denotes the set of TF neighbors connected to $j^{th}$ TG and $w_{ji}^{G-T}$ is the element of matrix ${\tilde{\boldsymbol{A}}}_{n}^{G-T}$. The updated TG representation is computed as:

$$\begin{aligned} \boldsymbol{h}_{j}^{G}=\beta_{j}^{self}\cdot{\tilde{\boldsymbol{h}}}_{j}^{G-G}+\beta_{j}^{neigh}\cdot{\tilde{\boldsymbol{h}}}_{j}^{G-T},\#\left( 6 \right) \end{aligned}$$

where $\beta_{j}^{self} and \beta_{j}^{neigh}$ denote the attention weights assigned to the intrinsic TG feature and the upstream TF-derived regulatory information, respectively.

By substituting the neighborhood aggregation term, we obtain:

$$\boldsymbol{h}_{j}^{G}=\beta_{j}^{self}\cdot{\tilde{\boldsymbol{h}}}_{j}^{G-G}+\beta_{j}^{neigh}\cdot\sum_{i\in\mathcal{N}\left( j \right)} w_{ji}^{G-T}\cdot\boldsymbol{h}_{i}^{T}$$

$$\begin{aligned} =\beta_{j}^{self}\cdot{\tilde{\boldsymbol{h}}}_{j}^{G-G}+\sum_{i\in\mathcal{N}\left( j \right)} \left( \beta_{j}^{neigh}\cdot w_{ji}^{G-T} \right)\cdot\boldsymbol{h}_{i}^{T},\#\left( 7 \right) \end{aligned}$$

Accordingly, from the TG-side update, the effective contribution of the edge between $i^{th}$ TF and $j^{th}$ TG is defined as:

$$\begin{aligned} \tilde{w}_{ji}^{G-T}=\beta_{j}^{neigh}\cdot w_{ji}^{G-T},\#\left( 8 \right) \end{aligned}$$

This term reflects how strongly the $j^{th}$ TG relies on the upstream regulatory information from connected TFs in a specific cell.

**Reconstruction of cell-specific TF-TG regulatory strength**

The two quantities $\tilde{w}_{ij}^{\text{T}-\text{G}}$ and $\tilde{w}_{ji}^{G-T}$ describe the effective contribution of the same TF-TG edge from two complementary perspectives: the TF-side dependence on TG-neighborhood information and the TG-side dependence on upstream TF information. To incorporate both directions of message passing, we define the reconstructed edge-level contribution between $i^{th}$ TF and $j^{th}$ TG in a cell as:

$$\begin{aligned} \hat{w}_{ij}^{c}=\tilde{w}_{ij}^{\text{T}-\text{G}}+\tilde{w}_{ji}^{G-T},\#\left( 9 \right) \end{aligned}$$

For each cell, the reconstructed regulatory matrix is therefore defined as ${\hat{\boldsymbol{A}}}_{T-G}$. This matrix preserves the topology of the reference TRN through $\boldsymbol{A}^{T-G}$, while introducing cell-dependent regulatory weights through the learned attention coefficients. Therefore, ScanNet does not infer a completely new regulatory graph for each cell. Instead, it reweights the reference TF-TG network according to the cell-specific message-passing dependencies learned by the regulation-level encoder. The resulting matrix ${\hat{\boldsymbol{A}}}_{T-G}$ can be interpreted as a cell-specific reconstructed TRN and used for downstream analysis of regulatory heterogeneity.
